# Digital Twin for Continuous Production of Virus-like Particles toward Autonomous Operation

**DOI:** 10.1021/acsomega.4c04985

**Published:** 2024-07-25

**Authors:** Alina Hengelbrock, Finja Probst, Simon Baukmann, Alexander Uhl, Natalie Tschorn, Jörn Stitz, Axel Schmidt, Jochen Strube

**Affiliations:** † Institute for Separation and Process Technology, Clausthal University of Technology, Clausthal 38678, Zellerfeld, Germany; ‡ Faculty of Applied Natural Sciences, Technische Hochschule Köln, Leverkusen 51379, Germany

## Abstract

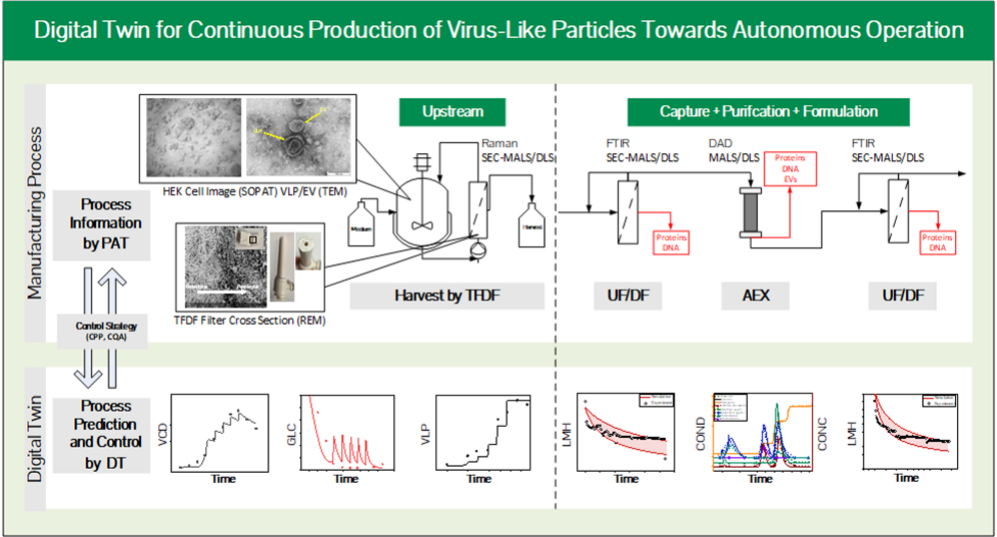

Lentiviral vector and virus-like particle (VLP) manufacturing have been published in fed-batch upstream and batch downstream modes before. Batch downstream and continuous upstream in perfusion mode were reported as well. This study exemplifies development and validation steps for a digital twin combining a physical-chemical-based mechanistic model for all unit operations with a process analytical technology strategy in order to show the efforts and benefits of autonomous operation approaches for manufacturing scale. As the general models are available from various other biologic manufacturing studies, the main step is model calibration for the human embryo kidney cell-based VLPs with experimental quantitative validation within the Quality-by-Design (QbD) approach, including risk assessment to define design and control space. For continuous operation in perfusion mode, the main challenge is the efficient separation of large particle manifolds for VLPs and cells, including cell debris, which is of similar size. Here, innovative tangential flow filtration operations are needed to avoid fast blocking with low mechanical stress pumps. A twofold increase of productivity was achieved using simulation case studies. This increase is similar to improvements previously described for other entities like plasmid DNAs, monoclonal antibodies (mAbs), and single-chain fragments of variability (scFv) fragments. The advantages of applying a digital twin for an advanced process control strategy have proven additional productivity gains of 20% at 99.9% reliability.

## 1 Introduction

Lentiviral vector (LV) and virus-like particle (VLP) vector manufacturing in fed-batch upstream and batch downstream mode was first reported in 2003,^1^ with VIRPAC/VRX496 from VIRxSys corporation being among the first systems utilized for the production of particulates for clinical studies.^2^ Producer cell systems are predominantly based on human embryonic kidney cells (HEK293). LVs and VLPs are most frequently derived from human immunodeficiency virus type 1 (HIV-1).

Batch downstream in most cases is achieved by normal flow depth filter harvest, nuclease treatment, ultrafiltration/diafiltration (UF/DF), and anion-exchange (AEX) chromatography as well as size-exclusion chromatography (SEC) for polishing with overall yields ranging from 20 to 40%.^3−9^

Continuous upstream by perfusion for other producer cell lines and virus system combinations was reported for duck embryo-derived EB66/Zika virus (ZV)^10^ and immortalized avian suspension cell line/influenza A virus (IAV)^11^ by the MPI group. No subsequent downstream was done, but stable perfusion without product retention was achieved for approximately 12–14 days for ZV and over 50 h post-infection (hpi) for IAV.

Among the first works toward continuous perfusion-based upstream for HEK293 and LV, however, was carried out around the same time by Lavado-Garcia et al. from the Cervera Group^12^ and the Viral Vectors and Vaccines Bioprocessing Group.^13−15^

Lavado-Garcia et al. applied the established ATF (alternating tangential flow filtration (TFF)) mode with hollow fiber membranes, and although a 0.5 μm MWCO (molecular weight cutoff) membrane was used, they observed complete product retention.^12^

The latter group realized perfusion by a so-called TFDF system, which is the combination of tubular depth filters and high TFF with low shear magnetic centrifugal pumps, did not observe any product retention, and could perform perfusion for over 6 days post-induction (dpi). They also performed semicontinuous downstream by batch normal flow depth filtration and cycling between three Mustang Q AEX membrane adsorbers.^15^ In conclusion, they found a factor of 13 higher productivity of the semicontinuous process compared to the batch alternative.

HIV1-based LVs, unlike the VLPs produced in this study, are commonly pseudotyped with the glycoproteins of the vesicular stomatitis virus (VSV-G), which is cytotoxic. Therefore, the production of HIV-1(VSV-G) LVs requires biosafety level 2 conditions. The VLPs described here were generated by a novel stable 293FMos1.Gag/Mos2S.Env producer cell line (Stitz Lab; unpublished data) expressing mosaic Gag and Env proteins. In brief, the cell line was established using the previously reported piggyBac-based transposon vectors,^16^ yet cotransfected with mRNA encoding for the hyperactive transposase (HyPBase)^17,18^ following the published protocol used for transposon vectors derived from Sleeping Beauty.^19^

Based on literature knowledge on the transition from fed batch to perfusion,^20^ initial tests were performed with standard ATF equipment and membranes comparable to those used in studies with CHO cells producing monoclonal antibodies (mAbs).^21^ The main difference influencing the performance of the perfusion process between monoclonals and VLPs is the size in relation to the cells, 11 nm for mAbs and 100–150 nm for LVs and VLPs. Cell debris are smaller than the host cells (10–15 μm) but can get as small as the particulates (100 nm). So, a wide filter cutoff (for product passage) and an efficient tangential flow removal of debris and cells are the main challenges. Dedicated experiments comparing the performance of normal flow depth filters and perfusion in ATF mode showed blocking behavior due to cell debris and insufficient particle removal, which led to TFF being investigated for proof of increased filter capacity by higher tangential flow.

### 1.1 Fundamentals and State-of-the-Art

Continuous biomanufacturing (CBM) of biopharmaceuticals offers significant advantages over traditional batch production. These include agility, flexibility, quality, cost savings, and social benefits.^22^ But the pharmaceutical industry still relies mainly on batch processes. This has led to regulatory agencies such as the FDA encouraging the introduction of continuous processes in pharmaceutical production.^23^ That lack of agility, flexibility, and robustness in pharmaceutical production is potentially a risk to public health, as production failures can lead to drug bottlenecks.^24^

A key advantage of continuous manufacturing is the ability to increase production volumes without the typical problems associated with batch size increases. This creates a more flexible approach to production.^25^ This is particularly crucial in situations where production needs to be increased quickly due to bottlenecks or emergencies.^26^ Traditional batch processes are prone to disturbances due to their globally distributed supply chains, whereas continuous bioproduction allows for regional and national manufacturing that can reduce this vulnerability.^27^

CBM enables the use of advanced process control (APC) that can both increase product quality and reduce initial investment costs.^28^ While batch processes can also benefit from such controls, a reliable control strategy is essential for the automated operation of continuous processes. In addition, CBM usually has a lower environmental impact and requires well-trained skilled personnel.^29,30^

Another potential advantage of CBP is the shortening of supply chains.^31^ The current batch production process often requires intermediate products to be stored in containers and transported worldwide to the next production site.^32^ With continuous manufacturing, however, production can be organized regionally or nationally, which can significantly shorten supply chains.^33^

One of the most challenging tasks in modern biotechnology is to develop and implement digital twins (DTs) for Quality-by-Design (QbD)-based process approaches. These approaches require flexible operating points within a proven acceptable range and automation through APC with process analytical technology (PAT). Compared with conventional process control based on offline analytics and inflexible process specifications, this approach is superior. In the field of drug substances in particular, VLPs have shown considerable potential as flexible vaccine platforms. VLPs based on HIV, such as HIV-1 Gag VLPs, are prominent. These can be made even more versatile by adding heterologous envelope proteins, such as the S protein of SARS-CoV-2. As these are enveloped VLPs, precise process control with minimal holding times is essential.^34^

HIV belongs to the retrovirus family and is a lentivirus that causes, upon infection, the acquired immunodeficiency syndrome (AIDS). Individuals with AIDS are more susceptible to fatal opportunistic infections. Despite an over 25 years long quest, no effective vaccine candidate has been developed yet, highlighting the need for more intensive research.^35,36^ VLPs offer a promising approach for the presentation of antigens.^37^ These multiprotein and membrane particles resemble real viruses in their structure and organization, but do not contain a viral genome, making them safe and nonreplicative.^38^ The Gag, Pol, and Env polyproteins of HIV are proteolytically processed to subunit proteins during particle maturation. However, VLPs can also remain immature HIV particles consisting of uncleaved Gag precursor proteins in the absence of the viral protease encoded by the pol gene. Compared with soluble antigens, VLPs are superior because they induce a stronger cellular and humoral immune reaction without additional adjuvants. Their particulate and repetitive structure enables efficient uptake by antigen-presenting cells and triggers both humoral and cellular immune responses.^39,40^

The production of HIV-based VLPs in mammalian cells, especially in suspension cultures, is complex as these enveloped nanoparticles are susceptible to shear forces, pH fluctuations, and osmotic pressure. To overcome these challenges, QbD methods link process parameters to product quality characteristics. QbD-based process development is increasingly becoming the standard in the biopharmaceutical industry and is required by regulatory authorities.^34,41,42^ A comprehensive control strategy is essential to achieving the desired quality target product profile. Validated process models can define design spaces to avoid out-of-specification (OOS) batches. By the development of a DT based on these models, APC can be achieved. The holistic QbD approach ensures that product quality remains consistent from development to production. Real-time predictions of quality attributes through process models enable continuous optimization, even after submission.^34^

To enable continuous production of VLPs, a bioreactor system is required that gently retains the shear-sensitive cells^11,43,44^ while removing the product from the reactor.^45^ Various systems are available for cell retention of animal cells, including membrane-based systems such as ATF and TFF as well as density difference-based systems such as settlers (inclined and acoustic) or hydrocyclones.^44,46^ ATF perfusion is the most widely used technology for cell retention in the continuous production of recombinant proteins such as antibodies.^21,47^ The size of HI-VLPs, typically between 100 and 150 nm,^48,49^ poses a unique challenge for membrane-based systems. These particles can clog the membranes^50^ and lead to product accumulation in the bioreactor.^11,51−55^ Despite the prevalence of ATF perfusion technology in the production of recombinant proteins, the processing of HI-VLPs requires specific adaptations to avoid these problems and ensure a problem-free production process.

For cell retention in the production of virus particles, the acoustic separator and an innovative combination of depth filter and hollow fiber module have proven successful.^11,14,15,45,54−60^ To accelerate cell settling in density-based separation, the g-force can be increased by an acoustic resonance field.^44,61^ The acoustic separator not only enables separation of the product but also removal of dead cells, host cell proteins, and double-stranded RNA, leading to higher cell densities.^11,44,61^ However, for scale-up and higher throughputs, increased input power is required to maintain separation efficiency.^44,61^ This can result in higher temperatures in the separator, necessitating efficient temperature control. In addition, the decreasing concentration of dissolved oxygen in the acoustic separator can affect cell viability.^11,44,61^

A macroporous tubular filter is preferred for size-dependent separation, as it ensures efficient cell retention and good product permeability.^14,15,45,54,55,57−59^ In contrast to the acoustic separator, a complete recirculation of the cells into the bioreactor is also possible since they do not pass the filter, and thus no cell loss occurs. In addition, the recirculation time is shorter, since no settling of the cells is required for cell separation.^11,62,63^

A macroporous tubular filter offers decisive advantages over an acoustic separator when it comes to perfusion. By avoiding the need for a cooling system and a complicated pump strategy as well as the complete retention of the cells, the tubular filter is more efficient and easier to operate.

### 1.2 Quality Assurance, Quality Control, and Quality-by-Design

QbD-based process development has established itself as a leading approach for the creation of new pharmaceutical products such as VLPs, plasmid DNA (pDNA), and fragments. This approach ensures consistently high product quality and enables process improvements to be made, even after approval. In contrast to established platform processes for mAbs, there is a lack of comparable standardized processes for these novel products, which highlights the importance of QbD-based development.^64−66^

The FDA (U.S. Food and Drug Administration), the EMA (European Medicines Agency), the ICH (International Council for Harmonization of Technical Requirements for Pharmaceuticals for Human Use), and various industry working groups have taken action and published a large number of guidelines. One prominent example is ICH-Q8 to Q13, which relates to QbD.^67−70^

The implementation of QbD principles in process development requires a validated design space that ensures a consistent quality. This design space can be developed either through experimental data or through a profound understanding of the process. Therefore, there is an increasing need for DTs in process development.

Predictive process models, which serve as DTs, are crucial tools for quantitatively defined and knowledge-based process optimization. They accelerate process development and, at the same time, contribute to the generation of process knowledge. These models reduce experimental effort, and their applicability remains beyond the original approved design space as they are based on physicochemical principles. However, it is important to ensure that any model used is at least as accurate and precise as the experiments it is intended to replace.^66,71^ One-factor-at-a-time studies can indicate which parameters should be considered in multivariate studies. The application of design-of-experiment (DoE) principles enables the creation of an experimental design to characterize the design space.

### 1.3 Process Analytical Technology and Process Strategies

Studies on continuous bioproduction have shown that it is possible to achieve high product quality and deliver biologics on time and reliably.^21,72−75^ The transition to continuous production suggests that the process should be automated.^76,77^ Although operating times for continuous processes can be relatively short compared to traditional methods such as bulk or petrochemical production (typically 2 weeks to 2 months), autonomous operation enables consistent product quality and maintains the operating state around an optimum through APC strategies^78^ The benefits include lower operating costs, reduced production effort,^79^ and significant savings in quality assurance (QA) through real-time release testing (RTRT).^80^ APC is based on DTs, which are based on validated process models and must be clearly validated in regulatory decision-making processes. These are combined with PAT to perform statistically based data analysis to develop process control strategies.

The integration of the DT into the continuous manufacturing process of biologics requires key technologies and concepts such as PAT and QbD.^81−83^ Most sensors, spectroscopic ones in particular, are based on chemometric calculations such as partial least-squares regression and principal component analysis, which are already widely used in the literature. However, there are also model-based sensors that can be based on mass and energy balances as well as extended Kalman filters. The implementation of these sensors is more time-consuming but provides a deeper understanding of the process as they are based on physicochemical principles.^75,84−87^

For automation in continuous bioproduction, DTs rely on online process data to feed the real-time updated information into the process models.^33,75,88^ In addition to basic process parameters such as pressure, conductivity, pH, and temperature, the concentrations of target components and key impurities are also required to ensure that the data captured by the DT is reliable. Spectroscopic technologies such as Raman, Fourier transform infrared (FTIR), UV–vis, fluorescence, and circular dichroism have proven to be suitable analytical methods for various biologic manufacturing processes.^33^

**Figure 1:**
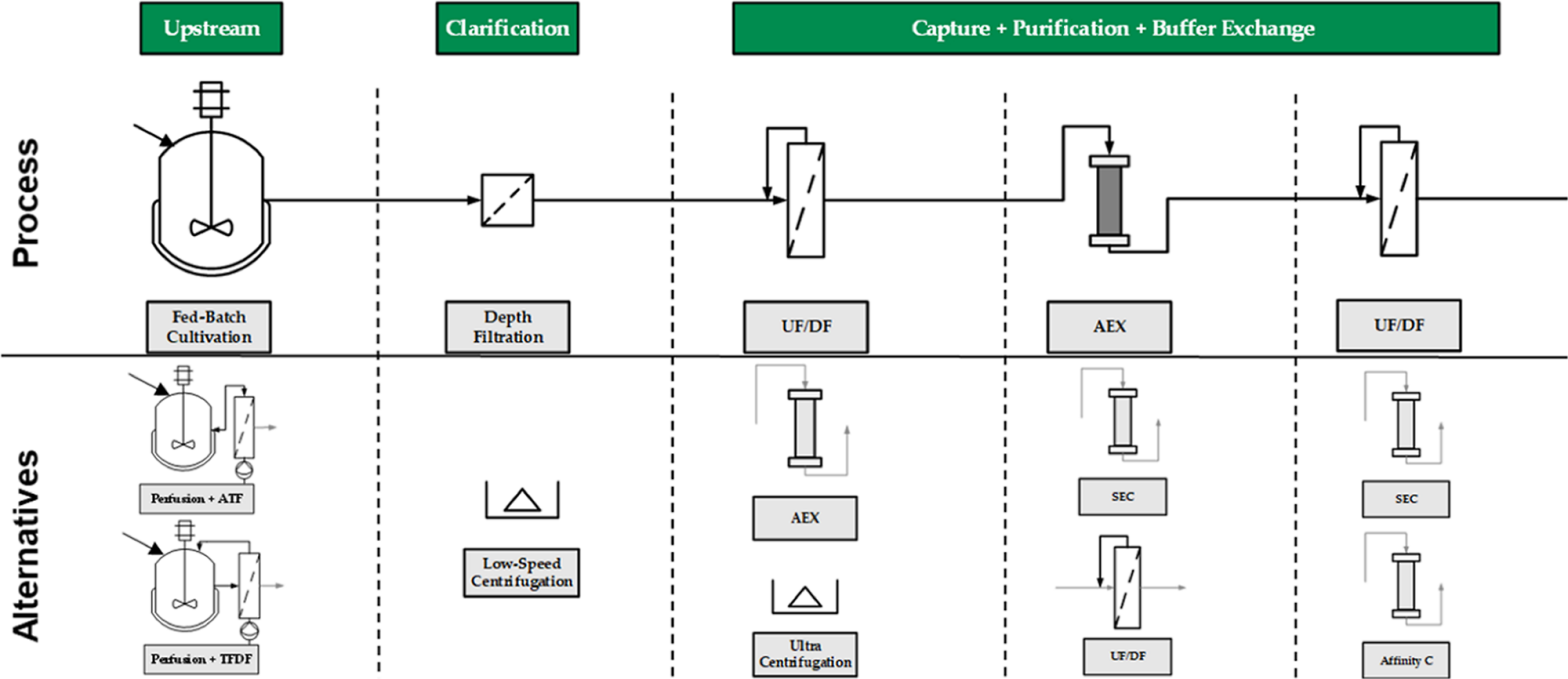
Overview of the HI-VLP batch production process and alternatives for continuous processing/harvesting and downstream process options.

The aim of this study was therefore to investigate alternatives to intensify batchwise production. After harvest via depth filtration or continuously using TFDF Technology, DF and initial purification were carried out using UF and DF. Further purification was performed by AEX chromatography, as shown in Figure 1. In order to meet the requirements of a QbD-based, automated process, a DT is needed. These receive data from the physical process in real time and can control the process by implementing suitable control strategies in order to maintain the product quality. They also make it possible to regulate fluctuations and disturbances in the process.^33,75^

## 2 Materials and Methods

### 2.1 Cultivation of HEK293 Cells

The VLPs investigated in this study were produced using the stable recombinant cell line HEK293FMos1.Gag/Mos2S.Env. This produces Gag proteins that are composed of mosaic epitopes derived from different HIV-1 variants. In addition, the cell line coexpresses mosaic envelope proteins.^16^

The cells were cultivated in Gibco Dynamis medium (Thermo Fischer Scientific, Waltham, USA) supplemented with 8 mM l-glutamine in a 2.5 L glass bioreactor (Sartorius, Göttingen, Germany) at 37 °C, pH 7, and a relative oxygen saturation of 40% based on air saturation. A segmented three-blade impeller with a blade pitch of 30° was used as the stirrer, which was operated constantly at 150 rpm. The cell concentration was determined once a day using the trypan blue exclusion method and a CEDEX XS (Roche Holding, Basel, Switzerland) for automatic cell counting. Glucose and lactate concentrations were determined daily from clarified cell culture samples by enzymatic-amperometric measurement using a LaboTRACE Compact (TRACE Analytics GmbH, Braunschweig, Germany).^55,89,90^

Cultivations were performed as fed batches for DSP process optimization. For this purpose, the HEK FS 2 feed (Sartorius AG, Göttingen, Germany) was started after 3–4 days when the glucose concentration had reached <2 g/L.

For possible continuous production of the VLPs, a perfusion was started after a fed-batch phase when approximately 17 million cells/mL were reached. Therefore, the setup described above was supplemented with a TFDF-30 filter (Repligen Corporation, Waltham, MA, USA) and a diaphragm pump. Perfusion was performed as ATF, with a set ATF frequency of approximately 0.5 L/min and a working volume of 850 mL. The volume flow of the continuous addition of the Dynamis medium and the removal of the product in the permeate were set to approximately 80 pL/cell/d for each cell.^55^ Based on the measured cell number, the required volume flows for the feed and the permeate were set using peristaltic pumps and monitored via the recorded masses of the feed addition and the permeate removal.

The different process modes are compared on the basis of the space-time yield (STY) and volumetric productivity (Pv). For a batchwise process, the total number of VLPs (*N*_total_) is calculated from the determined product concentration (*c*_STR,*t*_) and the reactor volume (*V*_STR_).^55^
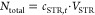
1

In a continuous process with a TFDF filter, the product is collected via the permeate. Consequently, the total product quantity is calculated from the concentration of VLPs in the permeate and the volume of permeate obtained.^55^
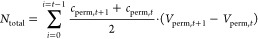
2

The STY describes the amount of VLPs produced in relation to the reactor volume and the overall process duration (*t*_total_).^55^
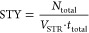
3

However, STY does not take into account the amount of medium consumed, which is the reason volumetric productivity is also considered for comparison. In the case of perfusion, the total amount of medium consumed takes into account the volumes of permeate and cell bleed in addition to the working volume in the reactor.^55^
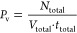
4

### 2.2 Harvest of VLPs

#### 2.2.1 Depth Filtration

When harvesting the culture broth batchwise, 210 mL of the resulting culture broth was depth-filtered with a Milistak+ D0HC (cutoff: 0.55–9 μm, Merck KGaA, Darmstadt, Germany). For the characterization of the depth filtrations, the depth filters Milistak+ CE25 (cutoff: 4–8 μm) and Milistak+ DE40 (cutoff: 0.55–1 μm) (both: Merck KGaA, Darmstadt, Germany) were connected in series to represent the two layers of the D0HC. The filtration was operated at a constant LMH of 107 ± 15 L/m^2^/h, and the pressure was monitored upstream and downstream of the filters.

#### 2.2.2 TFDF in ATF Mode

The ATF rate was set to 0.5 L/min. A peristaltic pump at the permeate side was used to initially set a flow of approximately 1 mL/min, which corresponds to the perfusion rate that would have to be set at the start of perfusion after the preceding fed batch (see Section 3.1). The setup described above was supplemented by pressure sensors on the feed, retentate, and permeate sides.

#### 2.2.3 TFDF in TFF Mode

For a further harvest of a second fed batch, the peristaltic pump was replaced by a membrane pump (QuattroFlow 150S, QuattroFlow Fluid Systems GmbH & Co. KG, Hardegsen, Germany) in order to reduce the shear forces acting on the cells and the product and to be able to set a recirculation rate of 1–2 L/min.^14,60^ In addition, the permeate side peristaltic pump was removed, and the TMP was manually increased to a maximum of 0.3 bar via a valve when the LMH decreased. The harvest was divided into three sections: first, a concentration by a factor of 1.6 was performed, followed by washing with 0.6 DF volumes of Dynamis culture medium, and finally, another concentration by a factor of 1.6.^14^

#### 2.2.4 Determination of Blocking Mechanism

The blocking of depth filtration and harvesting in ATF mode was determined by linear regression of the four main blocking mechanisms (cake, standard, intermediate, and complete) for dead-end filtration for constant flux (depth filtration) and constant pressure (ATF).^91,92^

When operating the filter in a tangential flow, the separation of particles due to the cross-flow must be considered in addition.^93−95^ Based on the single physical equation derived by Hermia, which makes it possible to establish a connection between the four blocking mechanisms, the blocking mechanism for the TFF was determined via the index *n* (*n* = 2: complete, *n* = 1.5: pore filling/standard, *n* = 1: intermediate, and *n* = 0: cake) and the consideration of cross-flow removal from the surface of the membrane.^93,95,96^

### 2.3 UF and DF

The initial product purification and concentration for the subsequent chromatography step were performed with a Sartorius SARTOFLOW Slice 200 benchtop system (Sartorius, Göttingen, Germany). A hollow fiber module with a cutoff of 300 kDa (Explorer12 ReUse 0.5 mm, Sartorius AG, Göttingen, Germany) was used. The starting medium was the cultivation broth harvested at the end of the fed-batch cultures, which was pooled and clarified by depth filtration. After a concentration by a factor of 3, a buffer exchange was performed with seven DF volumes, corresponding to a residual salt content of 0.8%, to MPA wAEX buffer (weak AEX chromatography). The experiment was performed at a transmembrane pressure of 0.5 bar and a shear rate of 3738 s^–1^.^89^

### 2.4 AEX Chromatography

After concentration and DF, the retentate was loaded onto a wAEX (5 mL, Poros GoPure D50, Thermo Fisher Scientific Inc., Waltham, MA, USA) without further sample preparation. The mobile phase A of the wAEX was 50 mM phosphate buffer with 5% sucrose and 2 mM magnesium chloride at pH 6. The elution buffer (MPB) contained additionally 1 M NaCl.^97−100^

The column was equilibrated with 10 column volumes of MPA. The UF/DF product was then loaded onto the column. Elution was performed in three steps. The first is at 20% MPB to elute initial impurities such as proteins, and the second is at 75% MPB to elute the product. Each step was held for 5 CV. Finally, the remainder was eluted with 10 CV 100% MPB.^97−100^ The flow rate was set to 0.26 CV/min.^97^ After elution, the column was equilibrated again with MPA for 10 CV. Disinfection was then carried out with 1 M NaOH for at least 10 CV. For fractionation, the UV absorbance was observed at 280 nm. In addition, the UV absorbance at 260 nm was recorded as a supplementary wavelength. The fractions from the flow-through, the wash (W), each elution step (E), and the disinfection (CIP) were sampled.

### 2.5 Analytical Methods

#### 2.5.1 p24 ELISA

p24 enzyme-linked immunosorbent assay (p24 ELISA) was used for the detection of VLPs. The VPK-107-H HIV p24 ELISA (Bio-Cat GmbH, Heidelberg, Germany) was performed according to the manufacturer’s instructions and analyzed at 450 nm with a multiplate reader (TriStar^2^, Berthold Technologies GmbH & Co. KG, Bad Wildbad, Germany). For the conversion of the resulting concentration, Gutiérrez-Granados calculated 3617 Gag monomers per VLP.^101^ Since immature HI-VLPs were produced in this study, the precursor protein p55 was present in the particles instead of the p24 capsid protein. As ELISA was designed to detect the p24 protein, this results in an underestimation of the p55 protein present. A correction factor of 10 was introduced to eliminate this. When converting the measured concentration of p24 to the particle concentration, the number of Gag monomers, the Avogadro constant, and the molecular weight must be taken into account. Due to the 2.3-fold greater molecular weight of the p55 protein in contrast to the p24 protein, the factor 2.3 was introduced as a further correction factor.^101^

#### 2.5.2 PicoGreen dsDNA Detection

The DNA concentration was quantified using the Quant-iTTM PicoGreen dsDNA Reagent Kit (Thermo Fisher Scientific, Waltham, USA) and performed according to the manufacturer’s instructions. The samples, diluted if the concentration exceeds the calibration range, were determined in duplicate at an excitation wavelength of 480 nm and a measured emission wavelength of 520 nm.

#### 2.5.3 Bradford Assay

The Pierce Bradford Protein Assay Kit (Thermo Fisher Scientific, Waltham, USA) was used to determine the total protein concentration and was carried out according to the manufacturer’s instructions using the microplate method. All samples were determined in duplicate and diluted if necessary. The evaluation was carried out by means of absorbance measurement at 595 nm. It should be noted that interferences can occur during the assay due to various substances, such as glucose or asparagine.

#### 2.5.4 SDS-PAGE

Sodium dodecyl sulfate-polyacrylamide gel electrophoresis (SDS-PAGE) gel images were generated for the qualitative determination of the proteins present and the p55 precursor protein. Premanufactured polyacrylamide gels (12% Mini-PROTEAN TGX Stain-Free Protein Gels, Bio-Rad, Germany) were used for this purpose. 8 μL of Laemmli buffer (ROTILoad 1, Carl Roth, Karlsruhe, Germany) was added to 25 μL of sample, and after an incubation time of 10 min at 96 °C, 20 μL of each sample was loaded onto the gel. In addition, 10 μL of a size standard (PageRuler 10–180 kDa, Thermo Scientific, Schwerte, Germany) was loaded. The electrophoresis was run for 30 min at 60 V and a further 60 min at 150 V. ROTIPHORESE10x (Carl Roth, Karlsruhe, Germany) buffer diluted 1 to 10 was used as a running buffer. The gel was then washed in ultrapure water for 30 min before being stained with ROTIBlue quick (Carl Roth, Karlsruhe, Germany).^102^

#### 2.5.5 Size-Exclusion Chromatography-Multiangle Light Scattering/Dynamic Light Scattering

Size-exclusion chromatography (SEC, TSKgel G5000PWXL, 7.8 × 300 mm; 10 μm; Tosoh Bioscience LLC, Montgomeryville, PA, USA) was used as an additional product analysis method. In addition to the DAD, a multiangle light scattering (MALS)/dynamic light scattering (DLS) detector (DAWN, Wyatt Technology, Santa Barbara, CA, USA) was used to obtain information on particle size and number. Separation was performed isocratically at 0.3 mL/min with a 25 mM sodium phosphate buffer at pH 8.^103^ The particle size and number were evaluated using ASTRA 8.1.1 software, where the VLPs are assumed to be spherical, monodisperse, and homogeneous with a refractive index of 1.46.^103,104^ The hydrodynamic radius was determined by using DLS. For this purpose, the software determines the diffusion coefficient from the time-dependent fluctuations in the scattered light by applying a second-order correlation function. Based on this, the hydrodynamic diameter was calculated, taking into account the eluent properties.^105,106^ The particle concentration is based on the light scattering, which was recorded by the MALS detector. To calculate the particle concentration, information about the refractive index of the particles and the solvent, as well as the particle volume, is required. The latter can be calculated using the radius, which is obtained by fitting the formula for spherical particles to the curve of excess Rayleigh ratios at the respective angles.^103,107−111^

#### 2.5.6 Nanoparticle Tracking Analysis

The particle concentration of the VLP samples was determined by nanoparticle tracking analysis (NTA) using a ZetaView 30x (Particle Metrix GmbH, Ammersee, Germany), at a wavelength of 520 nm. The bioreactor samples were centrifuged for 3 min, and 100*g* and the supernatant as well as the pellet, which was previously resuspended in medium, were measured.

Each sample was diluted with water to maintain the measurement range of 1 × 10^7^ to 1 × 10^8^ particles mL^–1^. The number determination was carried out over 5 cycles with a total image acquisition time of 60 s. Three recordings were made per sample.

#### 2.5.7 Dynamic Light Scattering

The particle sizes were determined using DLS measurements. The Malvern Zetasizer Nano ZS ZEN3600 (Malvern Panalytical Ltd., Malvern, UK) was used, and triplicate measurements were carried out at room temperature. The intensity distributions as well as the mean diameters of reproduced measurements were averaged, and the proportion of particles larger than 0.7 μm was determined by summation of the intensity curves for diameters > 0.7 μm. Measurements that were not comparable, e.g., due to particle sedimentation or poor mixing of the sample, were excluded. As in the case of the NTA, both the supernatant and the pellet were measured.

#### 2.5.8 Transmission electron microscopy

Transmission electron microscopy (TEM) was used to visualize the VLPs and extracellular vesicles (EVs), and negative staining was performed. The methodology was adapted from Rosengarten et al.^16^ In detail, the samples were mixed in a 1:1 ratio with a 2% (v/v) formaldehyde solution. Subsequently, 5 μL of this was applied to a carbon-coated copper mesh, and the mesh was incubated for 20 min at room temperature. After seven washing steps with phosphate-buffered saline (PBS) for 2 min each, the sample was fixed on the mesh with 1% (v/v) glutaraldehyde in PBS. After a further eight washing steps with deionized water, the sample was stained.^16^ As the uranyl acetate used by Rosengarten et al. is radioactive, it was replaced by a 4% (w/v) neodymium acetate solution and incubated in the dark for 4 min at room temperature.^16,112^

#### 2.5.9 Amino Acid Analytics

Amino acid concentration was measured by RP chromatography (InfinityLab Poroshell 120 HPH-C18; 3.0 × 100 mm; 2.7 μm; Agilent Technologies, Santa Clara, USA) and precolumn derivatization of amino acids with orthophthalaldehyde reagent in basic medium. If necessary, the samples were diluted beforehand to ensure complete derivatization. The column was tempered to 40 °C for better separation.

### 2.6 Modeling and Simulation

#### 2.6.1 Upstream

For modeling the intracellular metabolism of HEK293F suspension cells, the model published by Helgers et al.,^89^ which is based on the model for modeling the intracellular metabolism of CHO DG44 cells,^113^ was used as a starting point. The model consists of multiplicative Michaelis–Menten equations or variants derived from them, which represent the inhibition or activation of a reaction as a result of an accumulation of activator or inhibitor.^114,115^

The change in oxygen concentration in the bioreactor depends on the transfer of oxygen from the supplied air and the supplied pure oxygen into the medium as well as the consumption of oxygen during cell respiration. The transition of oxygen from the gas phase to the liquid phase is described using the two-film model and depends on the volumetric mass transfer coefficient *k*_L_*a* and the equilibrium concentration c_O_2__*.^116^
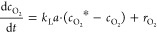
5

The equilibrium concentration of oxygen is described by Henry’s law, which takes the partial pressure of oxygen and Henry’s constant H into account.^116^
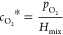
6

To determine the Henry constant of the medium (*H*_mix_), the influence of the medium components on the Henry constant of O_2_ in water (*H*_w_) is determined using eq 7. The media components to be considered here include sugars and alcohols. It is assumed that no alcohols are present in the medium and that the main sugar component is glucose. The influence of the medium components depends on the Sacharov constant *K*_*j*_ and the concentration of the medium component *c*_*j*_.^116^
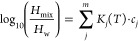
7

The temperature dependence of the Sacharov constant is calculated using eq 8 and that of the Henry constant of oxygen in water using eq 9.^116^
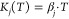
8
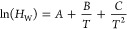
9

The *k*_L_*a* value depends on the one hand on the volume-related agitator power  and the gas velocity (*V*_g_). The coefficients *a*, *b*, and *c* used depend on the agitator geometry.^117^
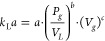
10

The gas empty tube speed is calculated using the supplied gas volume flow (*V*_g_) in relation to the area of the bioreactor (*A*_BR_).^117^
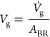
11

The change in the carbon dioxide concentration in the bioreactor is calculated in the same way as for the oxygen concentration. It is known from the literature that the volumetric mass transfer coefficient for CO_2_ is approximately 11% lower than that for oxygen.^118^
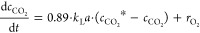
12
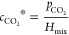
13

The change in pH due to the formation of CO_2_ and the addition of base is calculated using the Henderson–Hasselbalch equation^119^
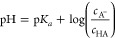
14

The energy balance of the bioreactor is shown in eq 15. This takes into account the energy input by the stirrer (*Q*_st_), the removal or supply of heat via the heat exchanger (*Q*_cool_) to ensure a constant temperature in the bioreactor, and the energy consumed by the metabolism. The stirrer power can be described by the dimensionless Newton number (Ne), the speed (*n*), the stirrer diameter (*d*_r_), and the density of the medium (ρ_S_). The heat output supplied or dissipated results from the jacket area (*A*_M_) of the bioreactor, the thermal conductivity (*k*_w_), and the temperature difference (Δ*T*) between the bioreactor temperature and the set point temperature. Energy is consumed during the reaction, which is described by the reaction enthalpy (Δ*H*_r_) and the rate of change of the concentration of oxygen through the reaction (*r*_O_2__). To determine the temperature gradient, the volume of the medium (*V*_s_) and the heat capacity (*c*_*p*_) are also taken into account.^115^
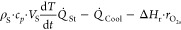
15
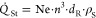
16
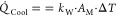
17

#### 2.6.2 UF and DF

The process model used is based on the research of Grote et al.^120^ Filtration is described using the Darcy–Weisbach equation^121−123^
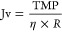
18

The main approaches for modeling flux decline in tangential flow UF are the resistance model, the gel concentration model, and the osmotic pressure model.^124^ Given the retentate stream, which is a suspension of VLPs, the resistance model provides the best description of the flux decline. Here, the total resistance *R* is represented in eq 18 as the sum of the initial membrane resistance *R*_m_ and the boundary layer resistance *R*_bl_, which is determined experimentally.^125,126^ The transmembrane pressure TMP is defined by eq 19
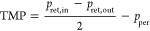
19

The model has been validated and applied for LV particles before by Hengelbrock et al.^84^ Model parameters have been checked to be appropriate for the experimental results, as discussed in Section 4.4.

#### 2.6.3 AEX Chromatography

The AEX chromatography was modeled using the model already published in Hengelbrock et al.^84^ It is based on the lumped pore diffusion model of chromatography in combination with Langmuir absorption.^127,128^ In order to achieve a more precise description of pore diffusion, the general rate model for chromatography according to eq 20 was used.^127^
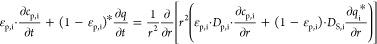
20

The parameters for this study were taken from the manufacturer’s document, and the Langmuir parameters were determined from the experimental data.

## 3 Results

### 3.1 Evaluation of Cell Retention Systems

During cultivation, which was carried out as a fed batch and then continued as perfusion in ATF mode, the filter was completely blocked after three hours of perfusion. This allowed 150 mL of permeate to be collected, which corresponds to a filter capacity of approximately 50 L/m^2^. Particle analyses were carried out to better understand the blocking and the material system used in this study. For a second harvest using TFF, the entire cell broth was harvested from a fed batch using the TFDF filter analogous to the literature.^14,60^

**Figure 2:**
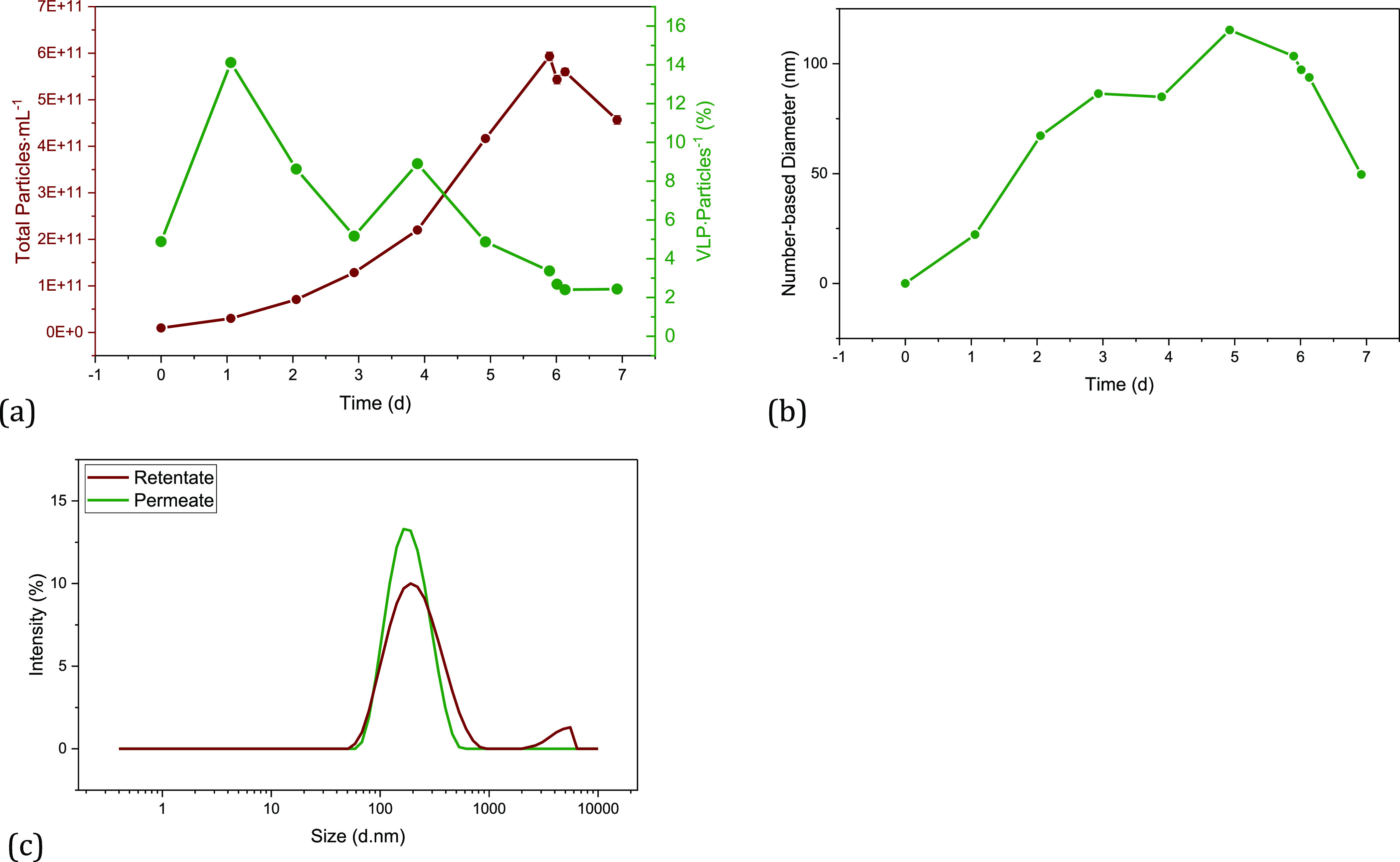
Results of the particle analysis of the fed-batch cultivation for the filtration experiments. (a) Total number of particles and percentage of VLPs in the total number of particles over the cultivation time, (b) number-based diameter over the cultivation time, and (c) intensity of particles over the particle size of retentate and permeate of a TFDF filtration.

As expected, during cultivation, the total number of particles in the medium increases over time (see Figure 2a). In addition, as the number of cells increases, more EVs are released into the medium, and the proportion of the product in the total number of particles decreases. Furthermore, an increase in the mean diameter can be observed over time (Figure 2b). This indicates that the proportion of larger vesicles and possibly aggregates of EVs and product increases.

The analysis of the particle size of the TFDF permeate shows (see Figure 2c) that all particles larger than approximately 700 nm are separated. As the filter module has a cutoff of 2–5 μm, the critical size range responsible for clogging the filter can be narrowed down to a range of 0.7–5 μm. About 10% of all particles are in this size range. Lorenzo et al. were able to demonstrate comparable particle size distributions in the cell broth.

#### 3.1.1 Batch Harvest via Depth Filtration

A depth filtration, which is used as harvest in the batch process, was carried out as a comparative test. The individual layers of Milistak+ D0HC (Sigma-Aldrich, St. Louis, USA) were used. The first layer (CE25) has a cutoff of 4 to 8 μm, and the second layer (DE40) has a cutoff of 0.55–1 μm.

**Figure 3:**
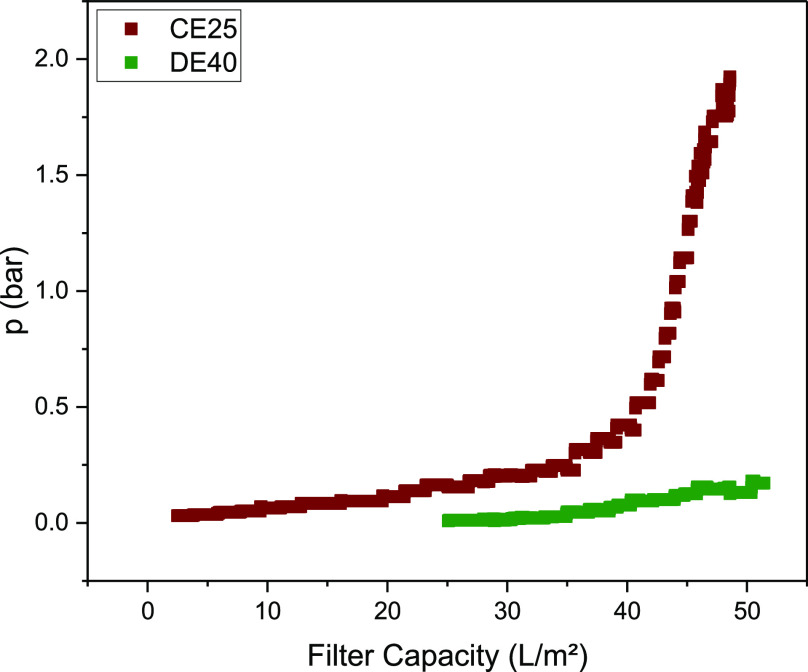
Pressure course over filter capacity for top layer (CE25, cutoff: 4–8 μm, brown dots) and bottom layer (DE40, cutoff: 0.6–1 μm, green dots) of Milistak+ D0HC depth filter.

The blocking of the first layer can be seen from the pressure curve (Figure 3a). The main blocking is therefore in the size range above 4 μm. As an increase in pressure also occurs within the second layer (Figure 3b), particles in the size range from 0.6 to 4 μm are also deposited. This is approximately 10% of the first layer. In total, therefore, 10% of the total particles are between 0.6 and 4 μm. The filter capacity is approximately 50 L m^-2^ and is consistent with the data from Helgers et al.^89^ for direct harvesting without preclarification. This is comparable to the observed filter capacity for continuous cultivation in ATF mode with a TFDF filter for cell retention. This suggests that the ATF mode behaves primarily like a direct flow filtration, and in contrast to classical hollow fiber modules, there is little or no backflow of the permeate through the pores, which should delay clogging of the filter. This is presumably due to the pore structure of a depth filter, which does not have a homogeneous structure but becomes narrower as the depth of the filter increases.

**Figure 4:**
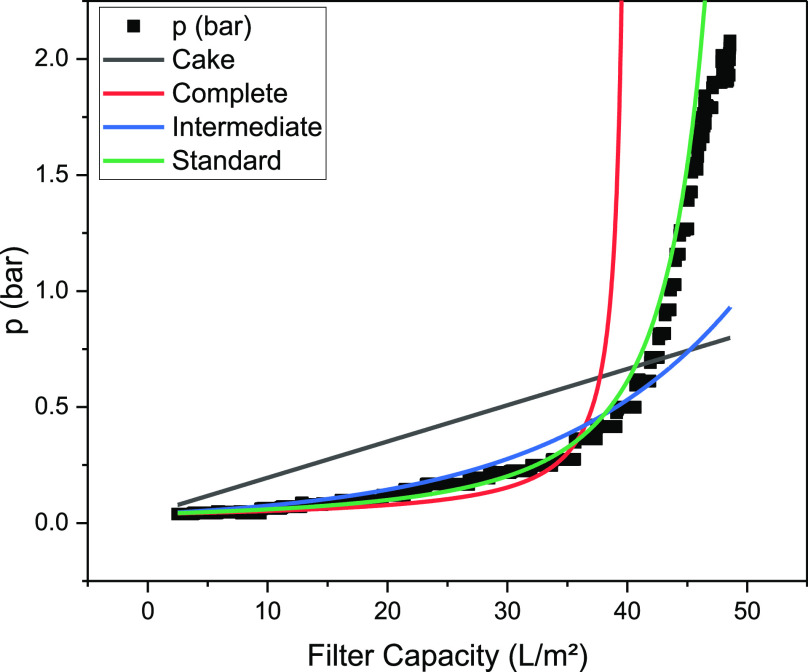
Pressure course (black dots) of harvest with Milistak+ depth filter D0HC with blocking constants determined by linear regression for cake (gray line), complete (red line), intermediate (blue line), and standard (green line) blocking mechanisms.

To determine the blocking mechanisms, the pressure curve was plotted for the four main blocking mechanisms of dead-end filtration in constant flux operating mode. A linear regression can be used to determine the dominant blocking mechanism and the blocking constant. With the help of the determined blocking constants, the pressure curves shown in Figure 4 are obtained.

The analysis shows that the increase in pressure cannot be adequately described by cake formation. Since the cell broth is a polydisperse mixture, the formation of a monodisperse cake layer is very unlikely. Only complete blocking without any occurrence of another blocking mechanism can also be assumed to be unsuitable as the filter materials used form heterogeneous states.

Based on the determination coefficient (see Table 1), the blocking mechanisms standard (*R*^2^ of 0.995) and intermediate (*R*^2^ of 0.975) can thus be identified as the main mechanisms present, which is consistent with previous harvests.^89^

**Table 1:** Regression Quality and Determined Blocking Constants of the Four Main Blocking Mechanisms for Batchwise Harvesting by Depth Filtration

blocking mechanism	*R*^2^	blocking constant
cake	0.638	0.003 ± 6.28 × 10^–5^ h/m^2^
complete	0.918	3.210 ± 0.014 1/h
intermediate	**0.975**	0.065 ± 2.78 × 10^–4^ 1/m
standard	**0.995**	0.037 ± 7.41 × 10^–5^ 1/m

#### 3.1.2 Cell Retention via the TFDF Filter in ATF Mode

**Figure 5:**
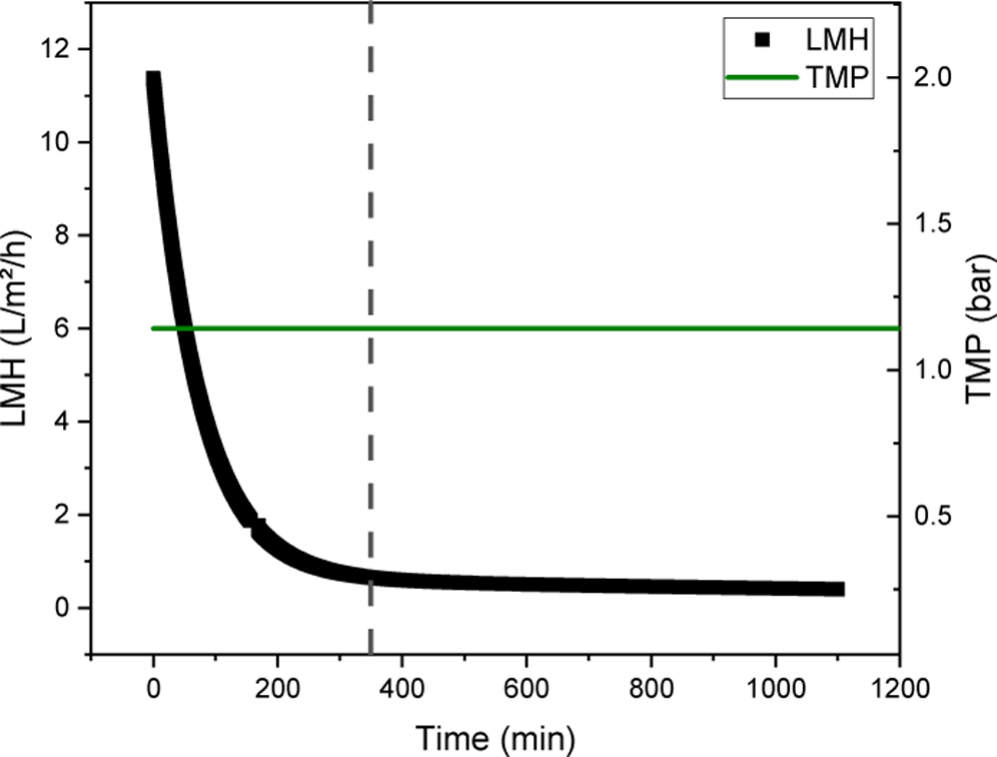
Progression of LMH and transmembrane pressure over the filtration time during harvesting using a TFDF filter in the ATF mode.

**Figure 6:**
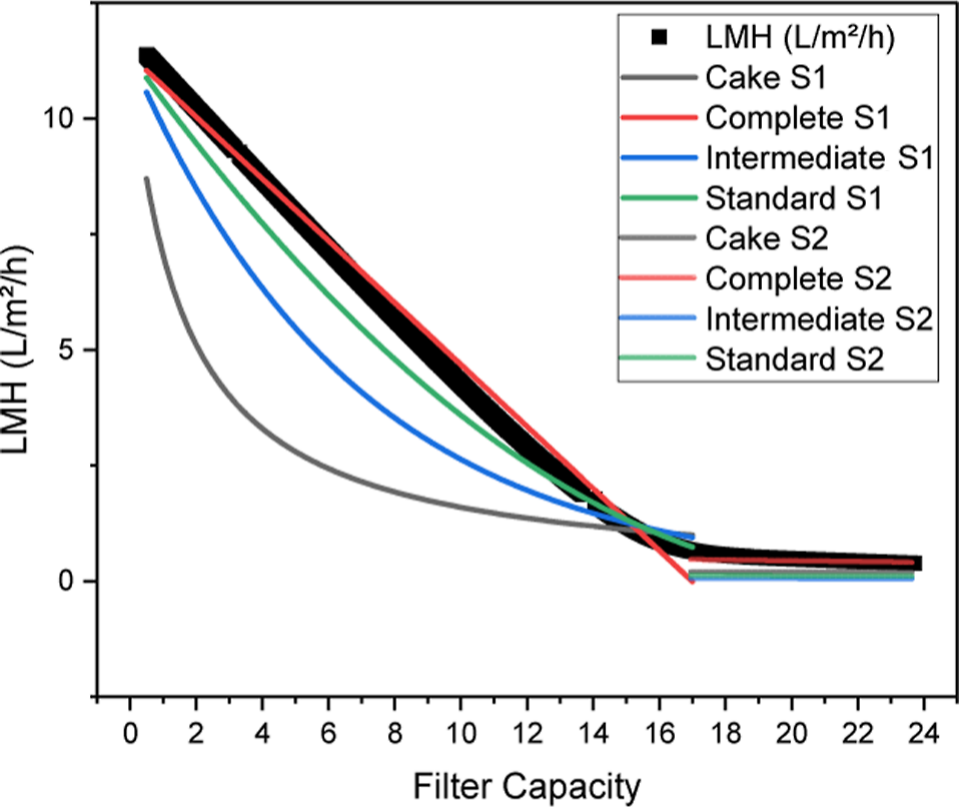
Course of LMH of harvest (black dots) with TFDF filter in ATF mode for a filter capacity <17 L/m^2^ (S1) and >17 L/m^2^ (S2, transparent colors); cake (gray line), complete (red line), intermediate (blue line), and standard (green line) blocking mechanisms.

For the ATF experiment, an ATF rate of approximately 0.5 L/min is used, and the permeate flow is set to 1 mL/min at the beginning. This results from the perfusion rate of 80 pL/cell/d, which is required for the targeted cell number of >17 million cells/mL at a working volume of 850 mL. This pump setting is maintained for the rest of the filtration process, so that the experiment is carried out at a constant TMP of approximately 1.1 bar (see Figure 5). The LMH decreases significantly at the beginning and decreases only slightly from minute 375 (corresponding to a filter capacity of 17 L/m^2^), whereby the permeate flow is already close to zero at this point (see Figure 5). Consequently, the evaluation was divided into two sections to determine the existing blocking mechanism from the decrease in the permeate flow: from the beginning of the experiment up to a filter capacity of 17 L/m^2^ (Figure 6) and from 17 L/m^2^ to the end. Due to the filter capacity of 50 L/m^2^ achieved in the perfusion, which corresponds to that of the harvest using depth filtration, it is assumed that the harvest using ATF and TFDF filters is primarily a direct flow filtration.

The blocking constants determined using linear regression led to the curves shown in Figure 6. The first section (<17 L/m^2^) can be well described with the complete blocking mechanism up to a filter capacity of approximately 15 L/m^2^ (*R*^2^ of 0.994). In the second section (>17 L/m^2^), there is no clear tendency toward a blocking mechanism. However, with the evaluation of the first section in the range of 15–17 L/m^2^, the experimental course can be well described by the standard blocking mechanism (*R*^2^ of 0.996). Consequently, particles are deposited both within the pores (>15 L/m^2^) and directly on the pores. In contrast to harvesting by depth filtration, no cake is formed, which indicates that particles >5 μm, such as cells, are successfully removed by the alternating tangential flow.

#### 3.1.3 Cell Retention via TFDF Filter in TFF Mode

Another fed batch was completely harvested by using a TFDF filter in TFF mode. In order to achieve feed flow rates of 1–2 L/min and to reduce the shear forces, a diaphragm pump was used. At lower feed flow rates, higher filter fouling is expected due to more inefficient removal of particles, leading to an increase in TMP and a decrease in product permeability^14^ and permeate flow. When the LMH decreased, the TMP was increased to a maximum of 0.3 bar on the retentate side (see Figure 8). The maximum TMP results from the maximum TMP recorded in the literature.^14,60^ In addition, a higher TMP is expected to cause more particles to be forced into the depth filter by the driving force of the pressure, which would lead to an accelerated decrease in the permeate flow and product permeability. After concentrating the cell broth by a factor of 1.6, 0.6 DV was exchanged, with the medium serving as a wash buffer. This was followed by a further concentration by a factor of 1.6, so that in the end, the same amount of permeate was removed as the feed.

**Figure 7:**
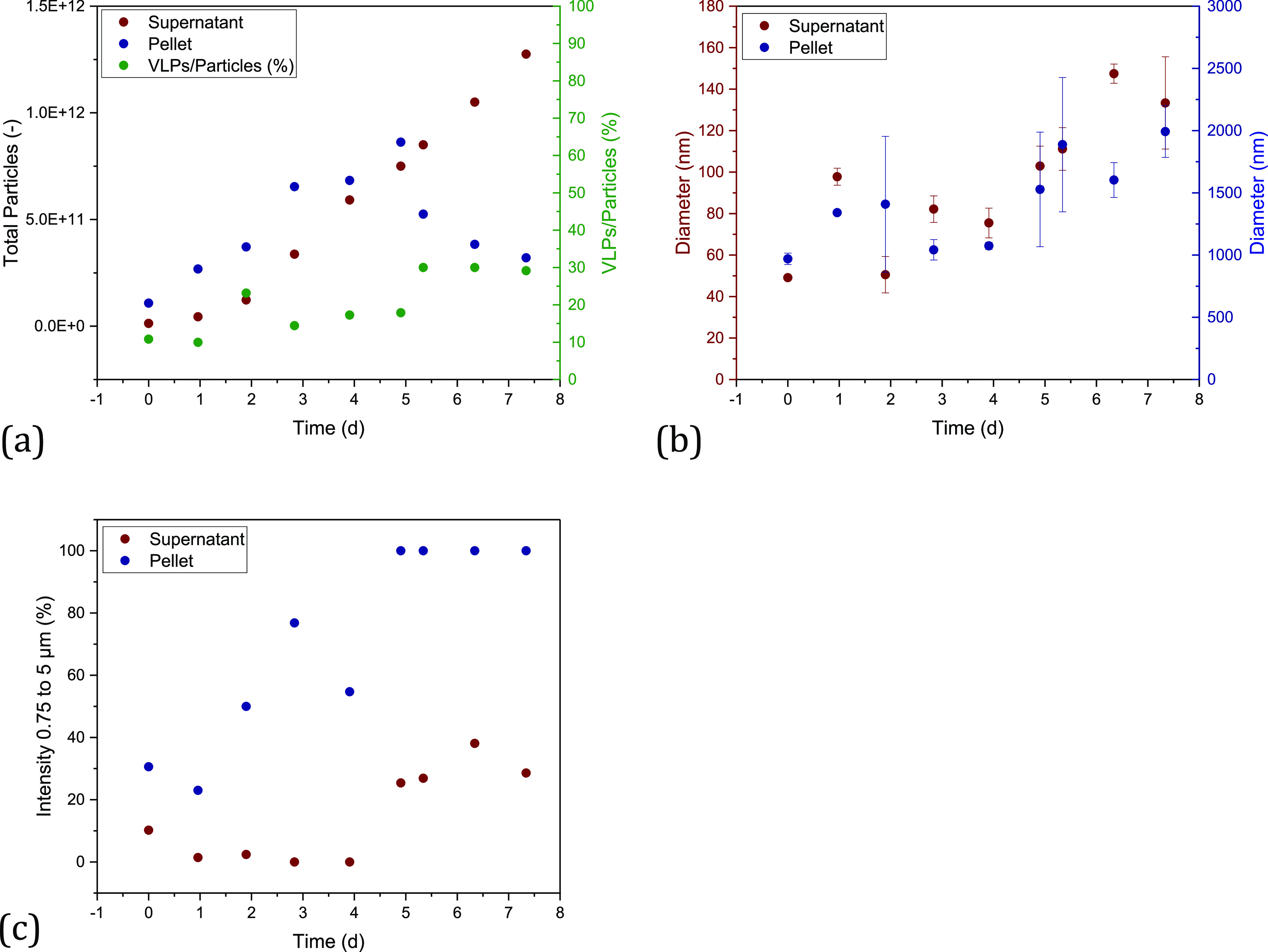
Result of the particle analysis of the fed batch for harvesting using TFDF in TFF mode. (a) Total number of particles in the pellet and supernatant of the bioreactor sample as well as the percentage of VLPs in relation to the total number of particles, (b) course of the number-based diameter in the pellet and supernatant of the bioreactor sample, and (c) percentage of particles in the supernatant and pellet, which are in the size range of 0.75–5 μm.

**Figure 8:**
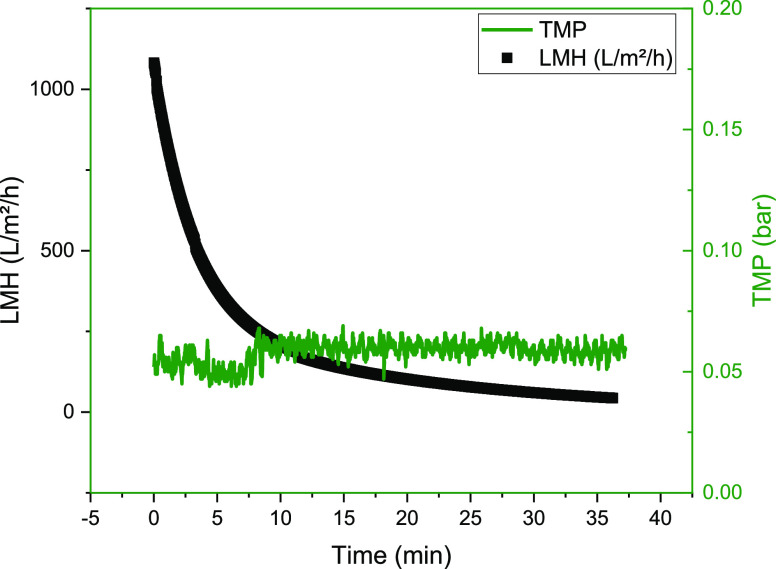
Progression of LMH and transmembrane pressure over the filtration time during harvest using a TFDF filter in TFF mode.

The total number of particles increases over the process time. In addition, the proportion of VLPs in the total number of particles also increases to 29% (Figure 7a). In the literature, proportions of 26–38% are documented for HI-VLPs produced by transient transfection.^5,129^ Moreover, the diameter in the supernatant as well as in the pellet increases over the process time, which indicates the increased production of EVs, e.g., caused by cell death. This also becomes clear when the critical size range for filtration is considered (0.7–5 μm). In the supernatant at the end of the fed batch, 29% of all measured particles are in this range (Figure 7c), which is 19% above the previously used fed batch.

At a recirculation rate of 1–2 L/min, a TMP of approximately 0.06 bar and a maximum LMH of 1082 L/m^2^/h is achieved, which drops to 43.6 L/m^2^/h during the first concentration (Figure 7).

**Figure 9:**
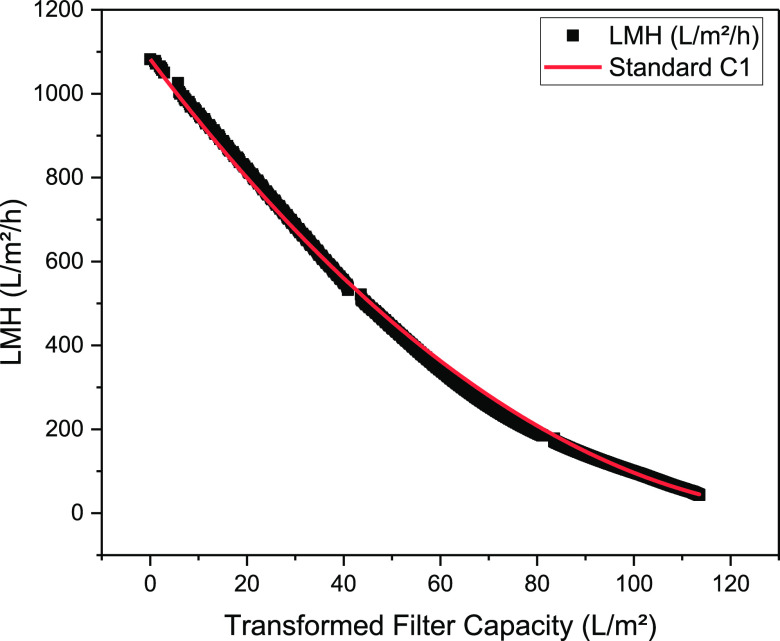
Course of LMH of harvest (black dots) with the TFDF filter in TTF mode for the first concentration step and the resulting course of the standard blocking mechanism.

After the linear regression of the four main blocking mechanisms, considering the removal due to the tangential flow, the standard blocking mechanism with a *R*^2^ of 0.998 can be identified as the present mechanism in the first concentration step (Table 2). The course of the resulting permeate flux for the standard blocking mechanism is shown in Figure 9. Consequently, the high recirculation rate prevents particles from being deposited on the pores and in their openings.

**Table 2:** Regression Quality and Determined Blocking Constants of the Four Main Blocking Mechanisms for Continuous Harvesting by TFDF in the TFF Mode

blocking mechanism	*R*^2^	*K*_n_	*J*_R_
cake	0.833	–2.22 × 10^–5^ ± 2.37 × 10^–7^	1515.25 ± 4.60
complete	0.996	14.33 ± 0.02 h	81.99 ± 0.49 L/m^2^/h
intermediate	0.950	0.01 ± 6.75 × 10^–5^	–269.80 ± 2.35
standard	**0.998**	0.23 ± 2.41 × 10^–4^	0

**Figure 10:**
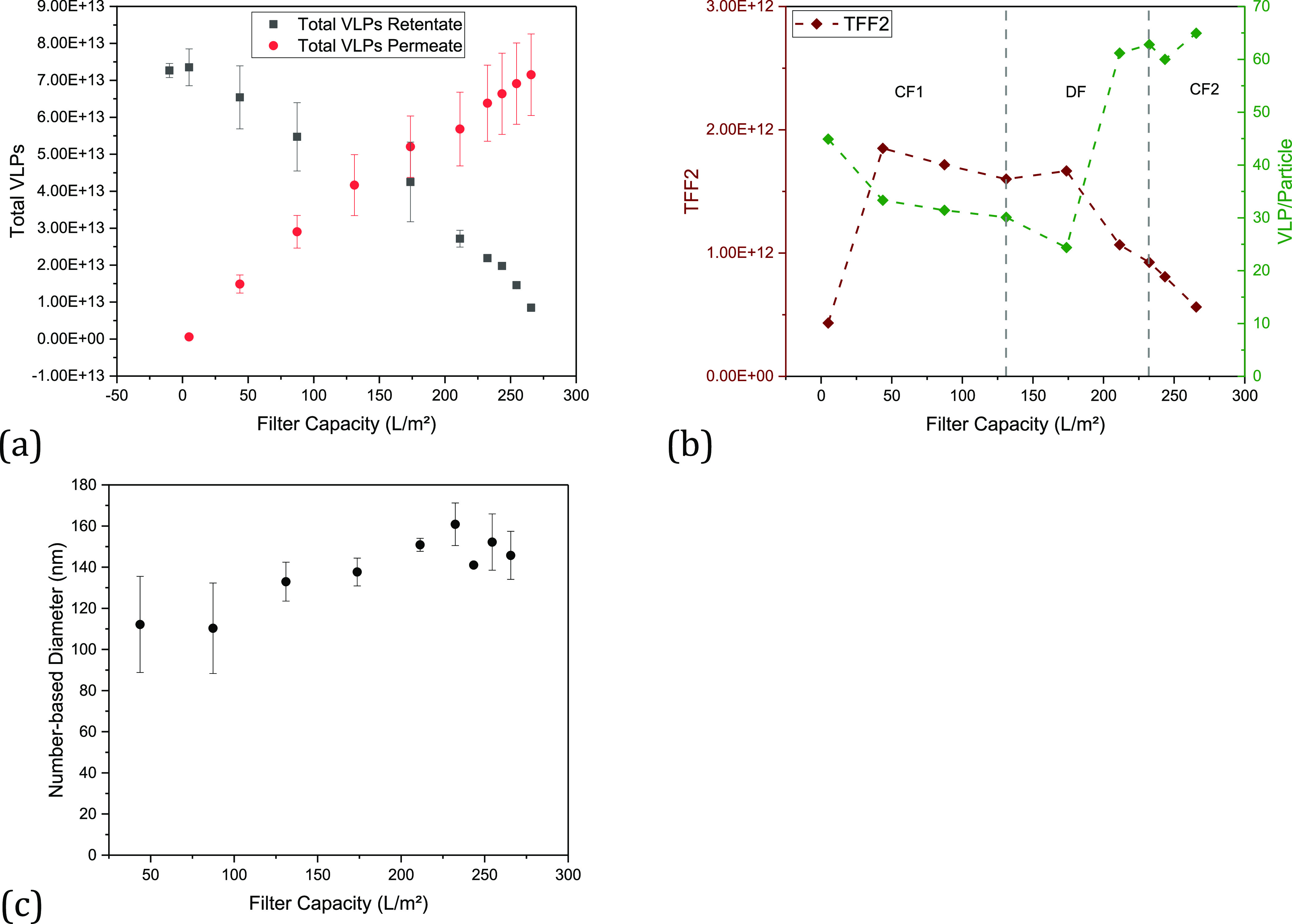
(a) Total number of VLPs in the retentate and permeate, (b) total number of particles and proportion of VLPs in the retentate and permeate, and (c) progression of the number-based diameter. Each plot was plotted against the filter capacity.

The diameter of the particles in the permeate increases slightly from the initial 112–146 nm during the test (see Figure 10c). The reason for this could be aggregates. Another cause could be the driving force of the TMP, so that more larger particles are pushed through the pores by increasing the TMP from 0.06 to 0.2 bar. During the entire experiment, blockage of the filter was only evident in the decrease in the permeate flow, which was approximately 100 L/m^2^/h at the end after increasing the TMP to 0.2 bar. A decrease in product permeability could not be observed, so that a recovery of 96% and a filter capacity of >266 L/m^2^ can be achieved (Figure 10a), while the proportion of VLPs in respect to the total number of particles increases to 65% (Figure 10b). In the literature, recoveries of 88–100% are reported for the harvest of LVs using the TFDF system.^14,60^

### 3.2 Process Mode Comparison

To compare the productivity of the different process modes, the STY and the volumetric productivity (Pv) are used. The batchwise production of HI-VLPs with batchwise harvesting using depth filtration and continuous harvesting using TFDF technology is compared with continuous production. In the latter, two cases are investigated, which differ in the start time of perfusion. In the first case, perfusion is started after the batch phase when at least 4 million cells/mL and a glucose concentration of <2.5 g/L are reached, as described in the literature.^55^ In the second case, a fed batch is first carried out until the target cell concentration of >17 million cells/mL is reached before perfusion is started. After reaching 20 million cells/mL, a bleed is introduced to maintain a constant viable cell concentration. Due to the higher inoculum concentration, perfusion is started 2 days earlier than in the literature, so that the total time is reduced to 16 days.^55^ For the perfusions, it is also assumed that the retention yield is identical to that of the continuous harvest.^130^

**Figure 11:**
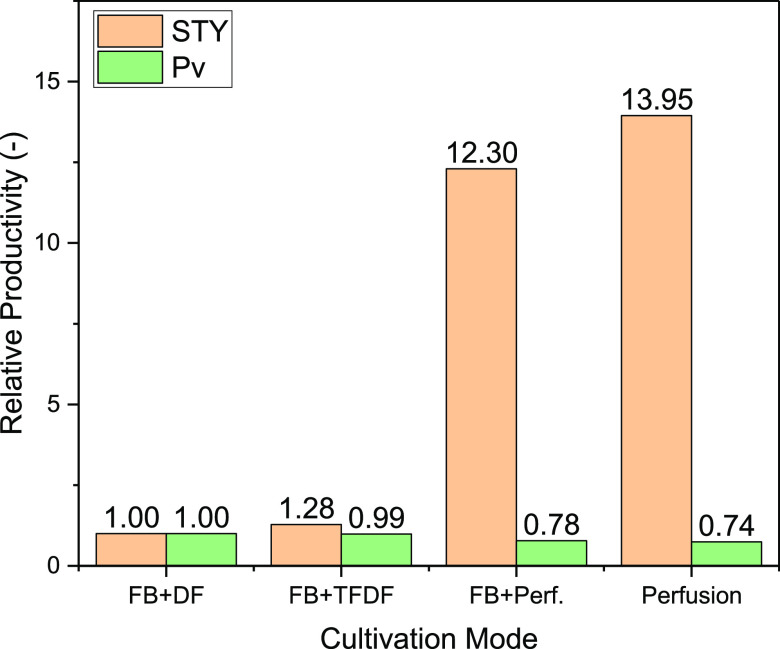
Relative STY and volumetric productivity of the different process modes in the USP in relation to the fed batch with depth filtration as the harvest.

The STY and Pv of the process modes in relation to the batch process are shown in Figure 11. The absolute values and process information are listed in Table 3. Due to the higher yield of the continuous harvest, an increase in STY of 28% is achieved, whereby the volumetric productivity is reduced by 1%. With a continuous USP, the STYs can be increased significantly by a factor of 12.3 (fed batch + perfusion) or 13.95 (perfusion). However, due to the higher media consumption, the volumetric productivity decreases by 22% (fed batch + perfusion) and 26% (perfusion).

**Table 3:** STY and Volumetric Productivity as well as Process Data for Different Process Modes in the USP

cultivation mode	fed batch	fed batch	fed batch + perfusion	perfusion
filtration technology	DF	TFDF	TFDF	TFDF
working volume (L)	1.14	1.14	0.85	0.85
cultivation time (d)	8	8	16	16
harvest volume (L)	1.14	1.14	13.45	16.06
maximum VCD (Mio. cells/mL)	20	20	20	20
total VLPs/batch	3.66 × 10^13^	3.66 × 10^13^	5.03 × 10^14^	5.71 × 10^14^
harvest/retention yield (%)	75	96	96	96
Pv (VLPs/mL/d)	3.01 × 10^9^	2.97 × 10^9^	2.34 × 10^9^	2.23 × 10^9^
STY (VLPs/mL/d)	3.01 × 10^9^	3.86 × 10^9^	3.70 × 10^10^	4.20 × 10^10^

The STY is significantly higher for continuous production than for the fed-batch processes considered. However, this does not take into account the entire media consumption; therefore, the volumetric productivity should be considered here. In order for the higher STY to compensate for the lower volumetric productivity, the TFDF filter must be permeable for the product over the entire process time. The perfusion processes for HI-VLPs published in the literature ran for up to 6 days.^13,15^ Although these were carried out with a stable cell line, product formation was started by induction and did not happen over the entire process time. Furthermore, Tona et al. detected filter fouling during the harvest after 3 days post-infection, so that the second concentration step had to be omitted.^14^ For the system of a continuously producing stable cell line used in this study, it therefore remains to be determined whether perfusion over the targeted 14 days is feasible without a loss of product permeability.

### 3.3 Process Summary

**Figure 12:**
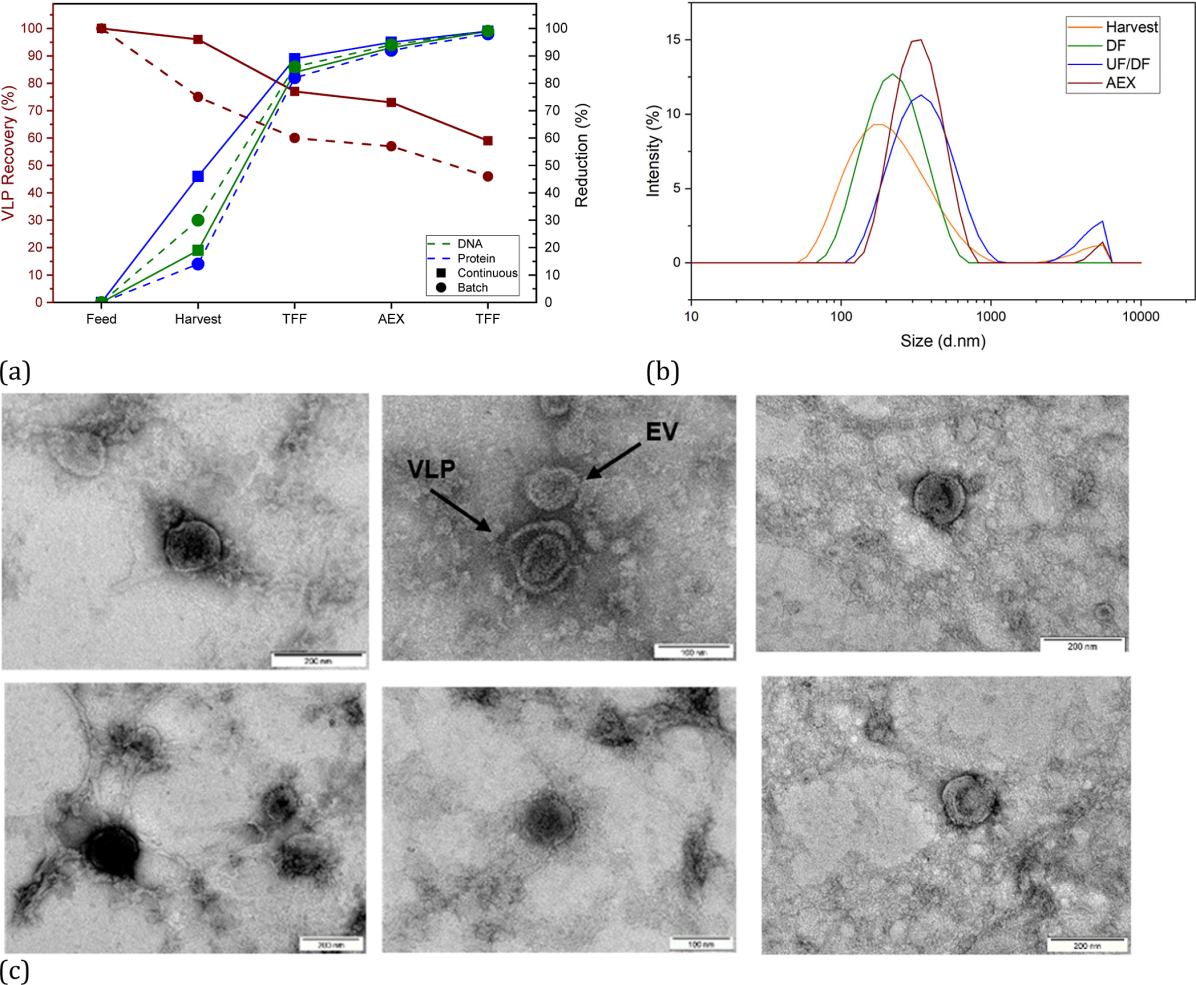
(a) VLP recovery (brown), total protein reduction (blue), and DNA reduction (green) over the process for a process with batch harvesting (DF, dots) and continuous harvesting (TFDF, squares); (b) intensities of the harvest (orange), the product from the depth filtration (green) as well as from the UF/DF (blue), and the product fraction from the w/AEX (brown) over the particle size; and (c) TEM images of samples after the harvest (left), after the TFF (center), and the AEX (right).

The most important parameters for the evaluation of the overall process are the VLP yields, as well as the protein and DNA reductions. These are shown in Table 4 and Figure 12a for the respective process steps for batch harvesting using depth filtration and continuous harvesting using the TFDF technology.

**Table 4:** VLP Recovery, Total Protein Reduction, and DNA Reduction over the Process for a Process with Batch Harvesting (DF) and Continuous Harvesting (TFDF)

unit	VLP recovery (%)		total protein reduction (%)		DNA reduction (%)	
	batch	continuous	batch	continuous	batch	continuous
bioreactor	100	100	0	0	0	0
harvest	75	96	14	46	30	19
TFF	60	77	82	89	86	84
AEX	57	73	92	95	94	93
TFF	46	59	98	99	99	99

Due to the 21% higher yield of continuous harvesting, a 1.28 times higher yield can be achieved for the overall process. In both processes, 98% (batch harvest) or 99% (continuous harvest) of the proteins and 99% of the DNA can be removed.

The samples of the respective stages are analyzed with regard to their size and percentage intensity, as shown in Figure 12b. The intensity curves correspond to the curves of Lorenzo et al.^129^ As described above, the particles in the size range from 700 nm to 5 μm make up approximately 10% of the total intensity that are separated during depth filtration. However, during concentration after UF/DF, particles >2 μm are again detectable, which may be due to aggregate formation during freezing and/or concentration during UF/DF. The intensity of the size range of the VLPs is increased by AEX. In addition, particles >800 nm are deposited, so that the intensity decreases significantly compared to the UF/DF retentate.

The qualitative detection of intact VLPs is analyzed with TEM at the various process steps (Figure 12c). In addition to the analysis of the samples directly after harvesting, the samples after the filtration step and after chromatography were also analyzed and are shown in Figure 12b. The VLPs are detected in the expected size range of 100 to 200 nm. In addition to the VLPs, EVs are also detected.

### 3.4 Digital Reproduction of Experimental Results

#### 3.4.1 Cultivation of HEK293 Cells

The previously published reduced metabolomic model was adapted to the new cell line and the modified cultivation medium and simulated for fed-batch cultivation.^115^

**Figure 13:**
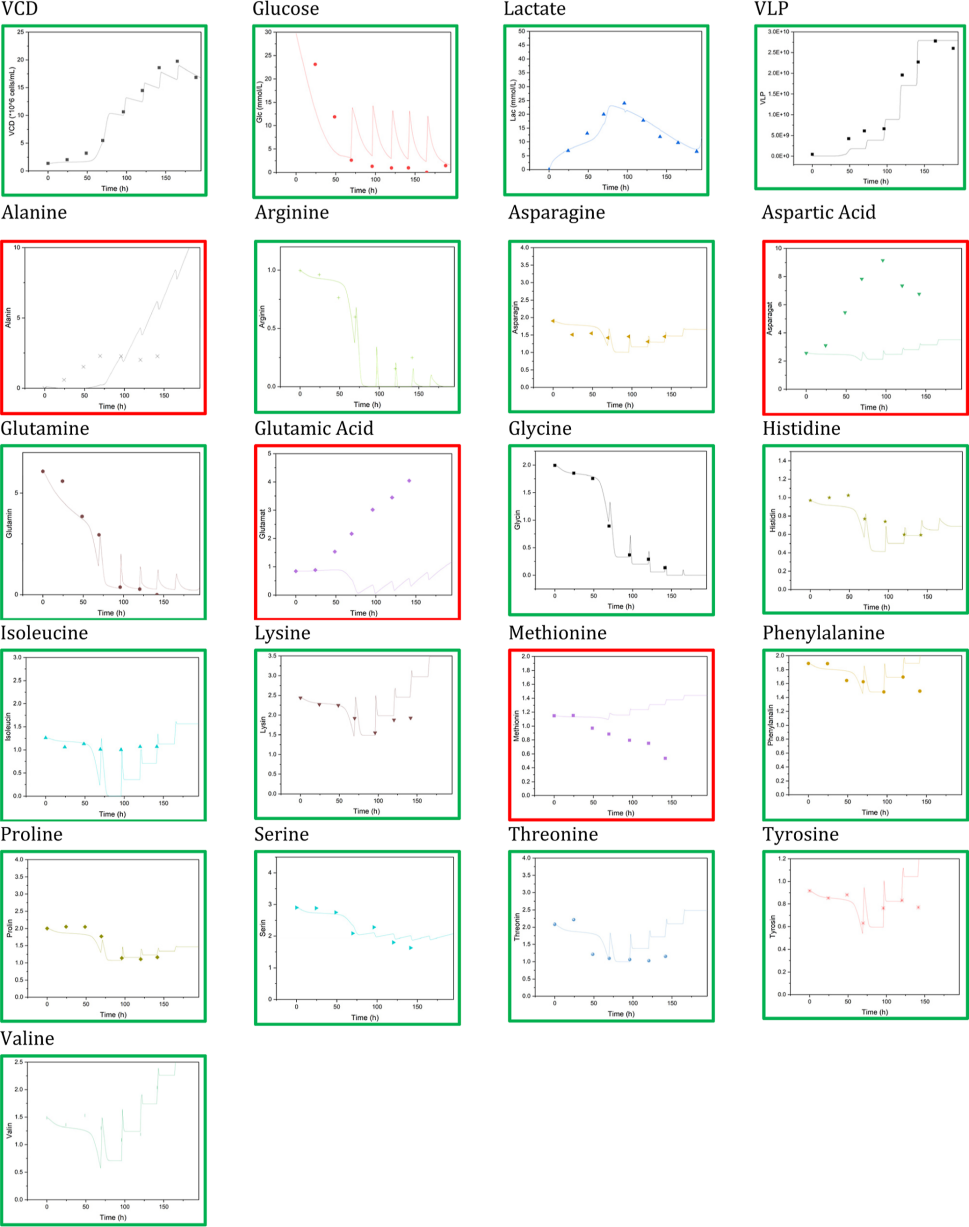
Simulated curves (lines) of amino acid (in mM), viable cell count (VCD, in million cells/mL), and product concentration (in VLPs/mL). Green boxes mark the simulated curves that match the experimental data (dots) well, and red boxes mark those that do not.

By adjusting the parameter set, the amino acid, glucose, lactate, product concentration, and viable cell density shown in Figure 13 could be calculated.

Of the 21 target substances considered, 17 are very well predicted.

To test the significance of all of the amino acids, a three-level factorial plan was simulated. For this purpose, the amino acid concentrations in the feed were reduced or increased by 50% in each case. The statistical evaluation of the experimental design is based on the reduction of the *p*-value. The maximum viable cell density as well as the process time, product concentration, doubling time, and cell-specific productivity at the maximum cell density are used as target variables. The values are set in relation to the experimental data. The significant parameters are the same for all of the other target variables.

**Figure 14:**
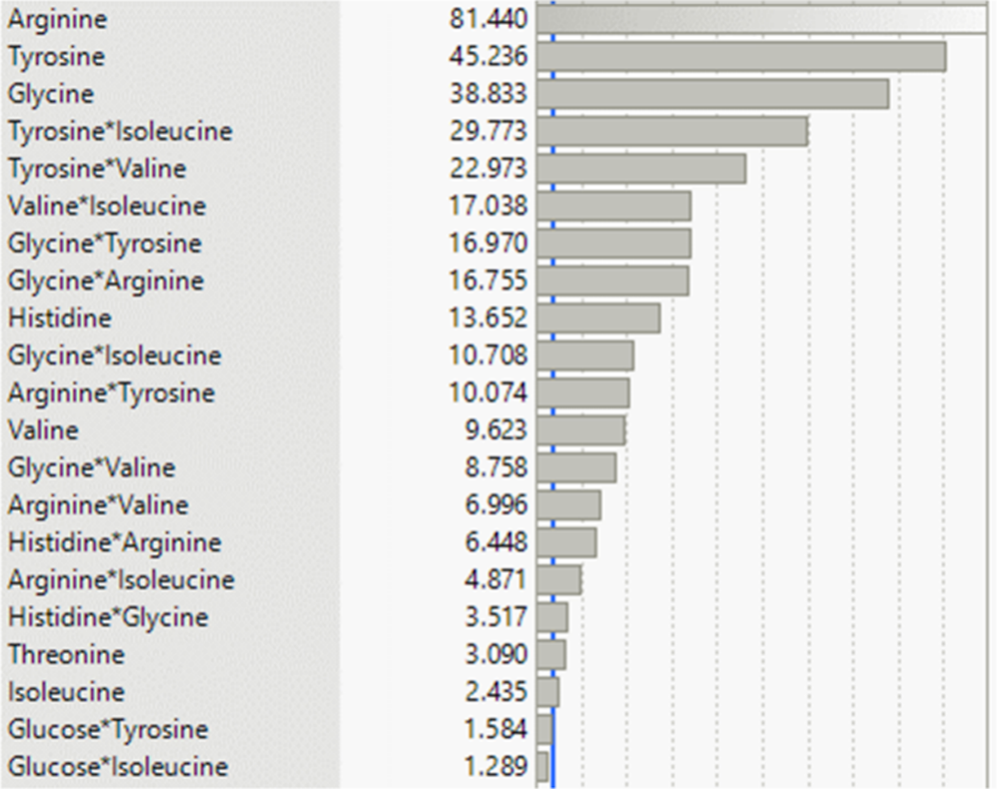
Log-worth (effect strength) of all significant amino acids (log-worth >2, blue line) on cell-specific productivity.

Figure 14 shows the parameters that have exceeded the significance line of the log value of two (blue line). Arginine, tyrosine, glycine, histidine, valine, threonine, and isoleucine can be identified as the most significant individual parameters (see Figure 14). The modeled concentration profile of these amino acids agrees well with the experimental data. The amino acids, which the model cannot reproduce with sufficient accuracy, are not significant for the investigated system. Thus, the model is able to reproduce the experimental data of the significant amino acids and is therefore considered validated.

For model-based optimization of feeding with regard to specific productivity, a three-level, factorial experimental design was simulated in which the time points (t1–t6) and the amount of feeding medium added (V1–V6) were each varied by 50% higher or lower based on the experimental values.

The statistical evaluation of the DoE with regard to the specific productivity in relation to the experimentally achieved specific productivity was carried out by stepwise reduction of the p-value. Outliers of the specific productivity downward (at approximately 0.3–0.7) and upward at >1.5, which result from unfavorable or beneficial combinations of feeding time and feed addition quantity, reduce the model quality to an *R*^2^ of 0.72. Most parameter combinations lead to a lower specific productivity than experimentally achieved (<1).

**Figure 15:**
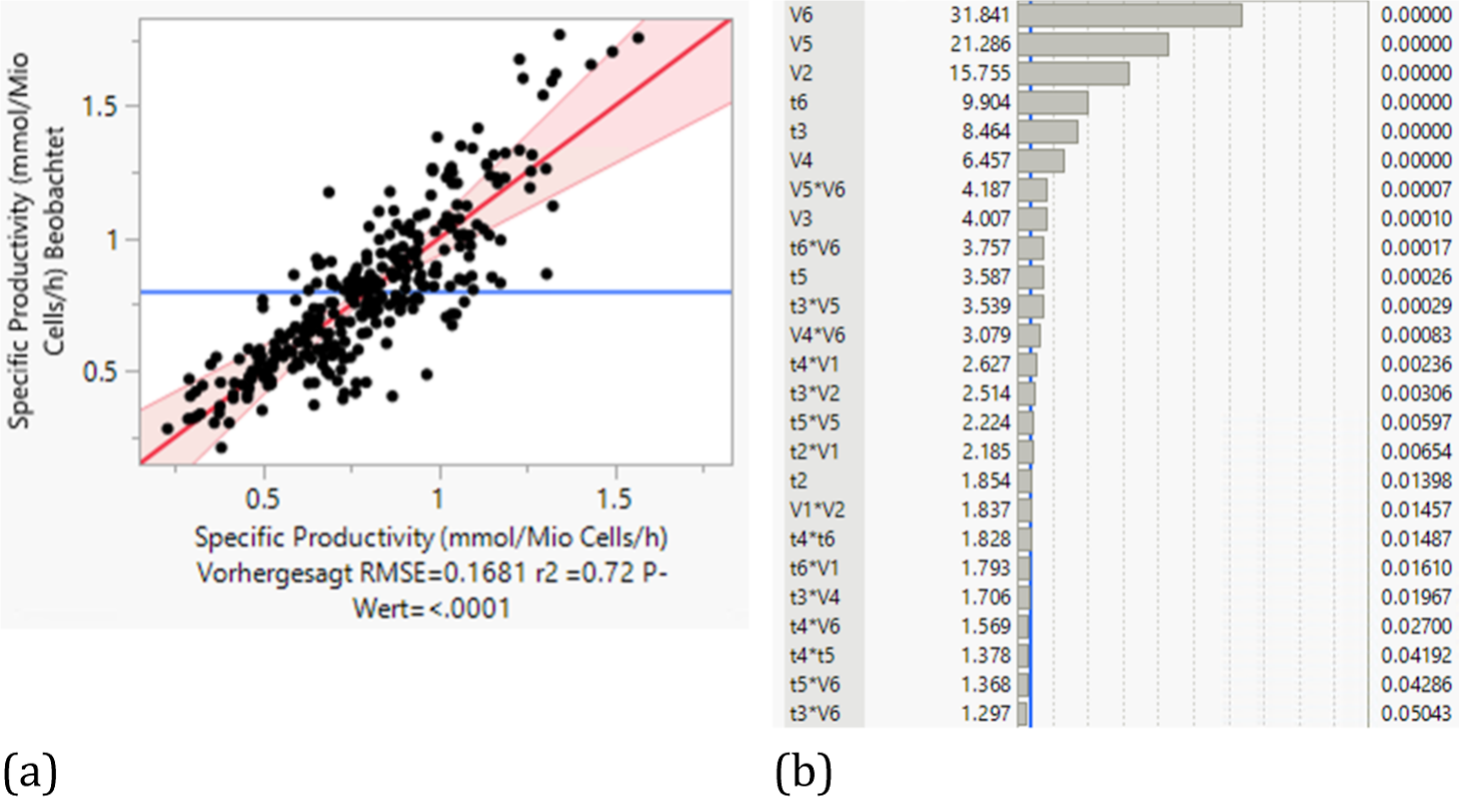
Regression and effect strength of feeding times and amounts on cell-specific productivity (a) actual vs predicted plot (b).

**Figure 16:**
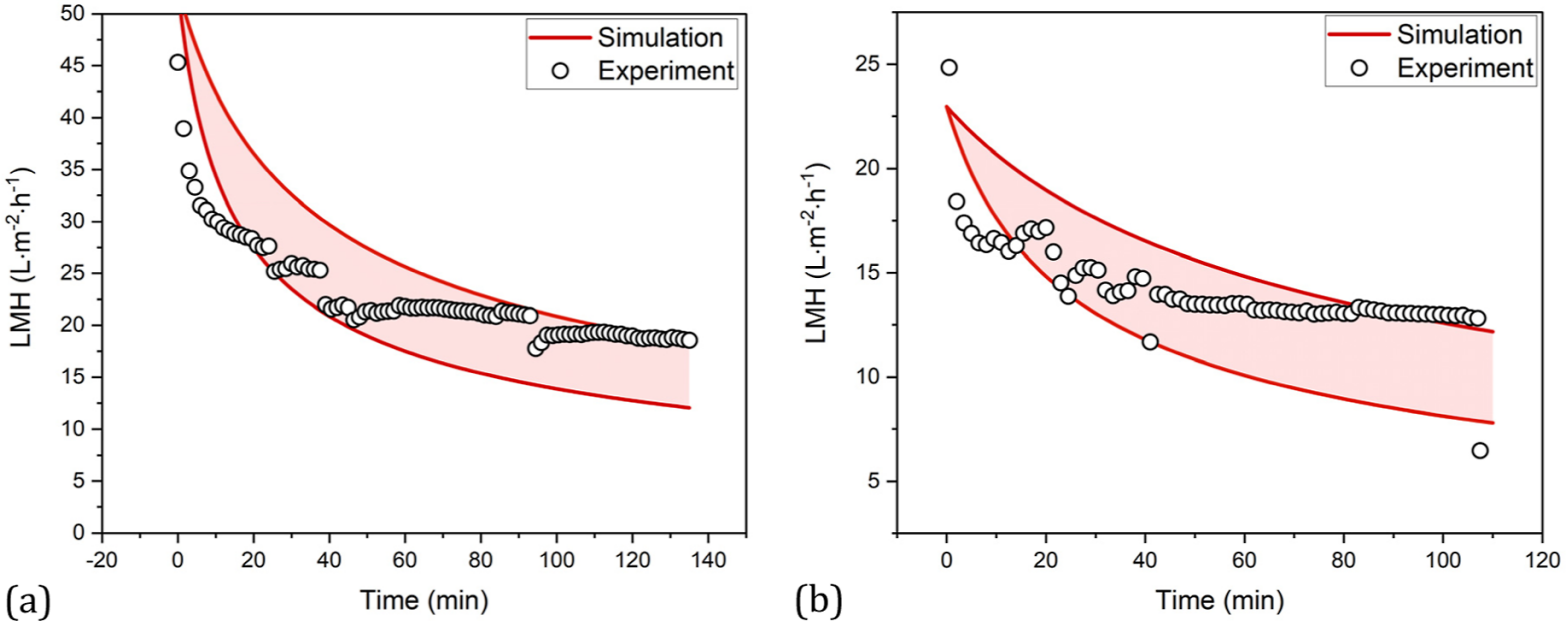
Comparison of the experimental and simulated curves of the LMH over time of UF (a) and DF (b).

From the significance list (Figure 15b), the trend can be seen in which the significance of the added feed quantity increases as the process progresses. The same applies to the feeding times, whereby the feeding time t4 is insignificant in the observed range, while the feeding time t3 is the second most significant parameter of the feeding times. If the optimal feeding strategy is predicted by the model, higher feeding quantities always lead to increased specific productivity in the investigated test range. However, even higher feeding rates lead to higher lactate formation, which influences cell growth and consequently productivity. For example, the specific productivity is reduced by over 50% when the feeding quantity is tripled. No clear statement can be made regarding the time points, although in the experiment conducted, some earlier feeding times would have increased the specific productivity (see Figure 16).

#### 3.4.2 Inline DF

The prediction of the DT for UF/DF agrees sufficiently well with the experimental results. The decrease in the LMH at the beginning of each experiment is described by the slow decrease in the flux resulting from the increase in the boundary layer resistance.

#### 3.4.3 AEX Chromatography

**Figure 17:**
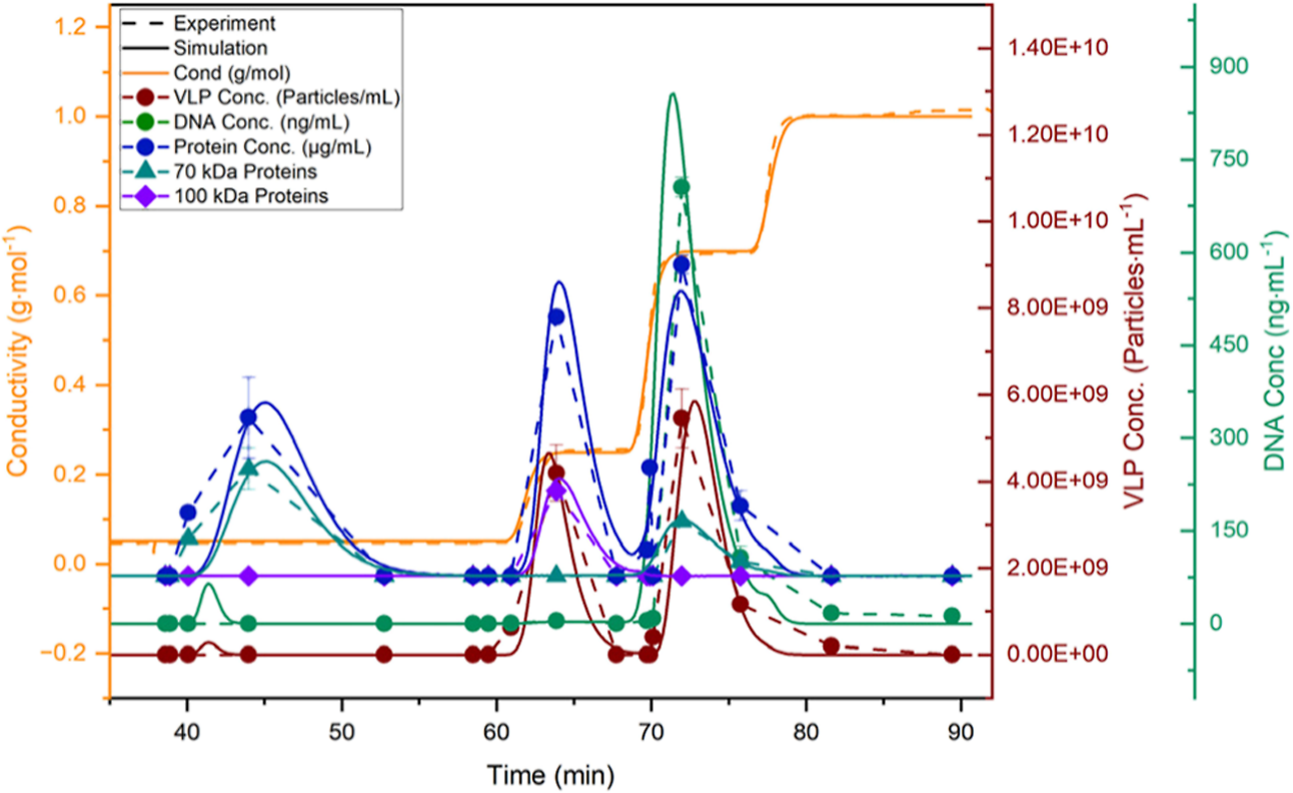
Comparison of the experimental and simulated chromatogram with regard to conductivity, pH value, DNA, protein, and VLP concentration of the w/AEX with a loading of 0.75 CV.

The model parameters for modeling the purification using AEX chromatography are determined experimentally. The axial dispersion coefficient and the voidage are determined fluid dynamically by measuring the electrical conductivity and the gradient. As the voidage is strongly dependent on the molecule, empirical values with similar columns are used as a guide. The data from Hengelbrock et al., which already refer to HIV Gag-VLPs,^84^ are used for this purpose. Fractionation is used to determine the concentrations and the isothermal parameters. The resulting simulation results are compared in Figure 17 with regard to the model parameters conductivity, VLP concentration, and DNA and protein concentration. Using the model, the curves are very well matched and lie within the experimental and simulative accuracy.

### 3.5 Process Control Strategy

To develop an autonomous operation system based on a validated DT including a PAT strategy under QbD approach, following work packages have to be carried out. First, a risk assessment including an initial Ishikawa analysis as well as subsequent OFAT and MFAT studies have to be performed. These studies result in a risk ranking, which summarizes the identified severity scores. This enables the definition of critical process parameters (CPPs), which are to be divided into well-controlled CPP (WC–CPP) and CPP, which are part of the final control strategy. In addition, a distinct experimentally validated DT is needed, and a PAT concept needs to be chosen. Success criteria are sufficiently fast and precise PAT-based determination of key process attributes (KPA). This workflow is already described in detail for different systems in the literature for continuous chromatography in PCC and CTCC mode^131−133^ as well as for membrane processes, especially SPTFF.^33,134−136^ These studies covered the same unit operations as used in this process, for batch and continuous operation. Validated process models and parameter determination workflows are published. The focus of this study is therefore the discussion of which control strategy for the different process steps is suitable.

**Figure 18:**
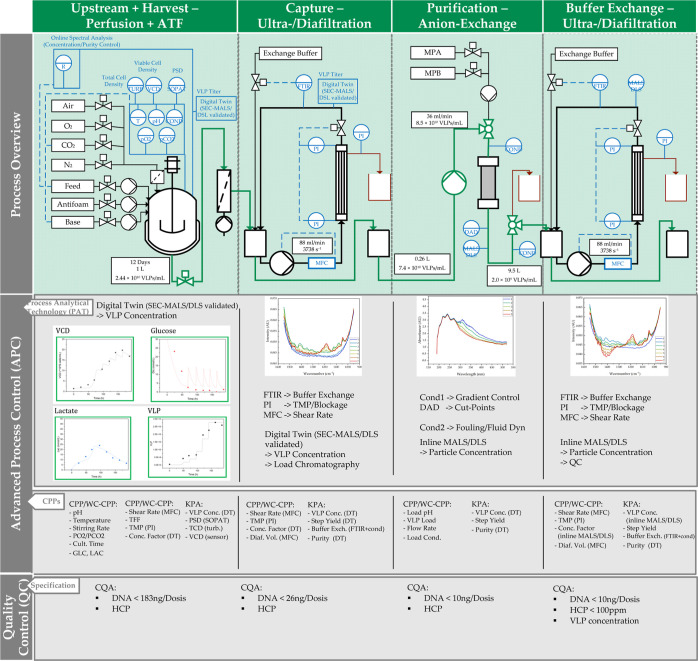
Process flowsheet with APC as a combination of DT and PAT.

Based on the flowsheet shown in Figure 18, which details CPPs and KPA as well as the chosen PAT detector, the control strategies are explained:

In upstream processing (USP), Raman and FTIR studies have proven feasible, and a Raman detector is chosen for glucose and lactate concentrations to enable an optimized feeding strategy. Turbidity determines TCD, and a sensor is feasible for the VCD measurement. In addition, a SOPAT system enables PSD of the cell system. As VLPs are represented by a heterogeneous large particle distribution, an offline SEC-MALS/DLS is needed for product concentration measurement at high particle amount and broad particle distribution, like cultivation with following filtration. Here, the DT is fed with the other online data and gives the VLP titer concentration, which has been validated by SEC-MALS/DLS offline. During USP, either product and impurity concentration, volume, or any combination can vary. Purity regarding DNA and HCP needs to be measured offline due to the low concentrations of the contaminants. Therefore, purity is online estimated with the aid of the DT proportionally of DNA and HCP to VCD/TCD ratio. The DSP to control these fluctuations is discussed with and without control strategy in batch mode (Table 5) and continuous mode (Table 6).

**Table 5:** Process Control Strategy Overview (Batch)

process variable	species	fluctuation	detection	unit operation	control mechanism	consequence of no control
volume		±10%	MFC/Balance	AEX	optimized loading	over- or underload
			USP-DT (SEC-MALS/DLS validated)			
USP concentration	VLP	+200%	USP-DT (SEC-MALS/DLS validated)	UFDF1	increase LMH to process a higher volume in the same time	AEX with proportional USP concentration increase factor overloaded → proportional factor as product loss
		>+200%	USP-DT (SEC-MALS/DLS validated)	UFDF1	increase LMH as above up to factor 2, then linearly longer process time to stay below critical flow, but productivity loss	AEX over factor 2 overload → over factor 2 product loss
		–50%	USP-DT (SEC-MALS/DLS validated)	AEX	linear longer load in the AEX	linear with concentration dilution Productivity loss in the AEX
	DNA	+600%	USP-DT	All	via constant separation factors in UFDF1, AEX, and UFDF2	robust up to factor 6
			QA (offline)			
		>+600%	USP-DT	UFDF2	linear prolonged UFDF2 operation	batch failure
			QA (offline)			

**Table 6:** Process Control Strategy Overview (Continuous)

process variable	species	fluctuation	detection	unit operation	control mechanism	consequence of no control
volume flow rate		±10%	MFC/Balance	SPTFF1	adjust LMH for constant flow rate in PCC	higher flow rate in PCC outside design space
			USP-DT (SEC-MALS/DLS validated)			
concentration	VLP	+10%	USP-DT (SEC-MALS/DLS validated) + DAD	PCC	adjust loading time by breakthrough detection	loss of product due to overloading
		–10%	USP-DT (SEC-MALS/DLS validated) + DAD	PCC	adjust loading time by breakthrough detection	loss of productivity
		>+10%	USP-DT (SEC-MALS/DLS validated) + DAD	PCC	redirect feed partially to surge tank	loss of product due to overloading
		<−10%	USP-DT (SEC-MALS/DLS validated) + DAD	PCC	adjust loading time by breakthrough detection	loss of productivity
	DNA	up to 600%	USP-DT	All	via constant separation factors in UFDF1, AEX and UFDF2	robust up to factor 6
			QA (offline)			

UFDF1 and 2 utilize PID standard controllers for TMP and LMH detected by PI and MFC as well as an FTIR detector for exact buffer exchange quality evaluation. CPP/WC–CPP in the UFDF are the shear rate, the TMP, the final product concentration, and the exchange volumes of the loading buffer. The shear rate is controlled via the MFC and kept within the control space. As the shear rate depends on the flow rate and therefore on the pump speed, this is a WC–CPP, which is unproblematic. The TMP is measured via the PI and kept within the control space. If an increase in TMP is observed, the control valve is opened. This is also a WC–CPP.

AEX is controlled via DAD on UV limits for fractionation cut points as event-based cut criteria. For the AEX, the amount of loaded mass is to be kept constant to ensure stable processing and scheduling. Therefore, the concentration and load volume need to be measured with DT (SEC-MALS/DLS validated) and MFC. This ensures the correct loading quantity for the AEX by proportional adaptation of the volume. If the concentration is lower than the process variation under consideration, the loading time and/or amount are increased accordingly with higher volumes.

#### 3.5.1 Batch Mode Concentration Control Scenarios

In batch processing, product concentration fluctuations detected by DT (SEC-MALS/DLS validated) up to an increase of factor 2 are controlled in the UFDF1 step. A typical UFDF process consists of the initial concentration phase, followed by DF and the final concentration.

To keep the concentration during the DF phase constant, proportional to the USP concentration fluctuation, less volume reduction is performed in the initial concentration phase. Hence, to achieve the same buffer exchange at the end of the DF phase, proportional buffer needs to be exchanged over time, resulting in an increased LMH. However, the LMH increase is limited by the critical flux value, which, in turn, is dependent on the particle concentration as well as the feed flow rate. For LV SPTFF, this has been investigated by Chaubal et al.,^137^ who found a critical flux of 41 LMH at 3E10 particles/mL at a feed flux of 55 LMH. As the normal operating point in batch UFDF1 is set to 20 LMH, increasing the LMH by a factor of 2 would be the upper limit. By this control strategy, a proportional larger product volume will be forwarded to the AEX step, which will need to adapt by additional cycles.

Alternatively, the increased USP concentration could be forwarded to AEX, whereas in the in-between UFDF1, the process parameters are unchanged from the reference values. This would result in a proportionally larger particle concentration during UFDF operation. If this strategy is chosen, the feed flux needs to be increased to counteract the increased fouling risk. This increase in the volume flow in return leads to shear stress and potential particle loss. The product volume forwarded in this approach will be constant; however, the increased concentration will be proportional to the USP fluctuation and needs to be handled in AEX by additional cycles.

So, when deciding between both strategies, the concentration should be adjusted in the UFDF step with a proportional larger product volume forwarded to the AEX step due to less fouling and shear stress in the UFDF.

#### 3.5.2 Volume Fluctuation Control Scenarios

In batch mode, the harvested volume can vary due to slight adjustments in feed media consumption. However, compared to the overall productivity of the producer cells, which might result in a concentration increase up to a factor of 2, the harvest volume should not vary more than ±10%. These volume changes can be well-controlled in UFDF1, which then results in a constant volume for the subsequent AEX and a proportional higher or lower VLP concentration.

#### 3.5.3 DNA Concentration Fluctuations

The DNA concentration levels expected for typical LV production platforms are less than <1 mg/L in upstream. This low concentration cannot be robustly detected by any PAT detector. Although most published process strategies involve an enzymatic digestion step with nuclease to handle DNA, the presented process does not introduce any additives to reduce analytical and process effort, securing the complete removal of such enzymes. To ensure that DNA is below the specification limit in the final product formulation (<10 ng/doses), the separation factors from UFDF1 to AEX and final UFDF2 are sufficiently large, such that in reference scenario, the DNA concentration is less than 1.5 ng/doses and up to a factor of 6, increased DNA impurity levels in USP are tolerable. For process control, the VLP purity regarding DNA and HCP is therefore determined with aid of DTs. Offline analysis is applied for QA.

If DNA levels after AEX exceed this threshold, then DNA levels can be further reduced by increasing the number of DF volumes in UFDF2.

#### 3.5.4 Continuous Mode Concentration Control Scenarios

As the real volumetric flow rate and concentration of the perfusion can change over time, the process must compensate for the fluctuation to ensure constant output and quality of the product. This is determined in the mass flow controller and regulated in the SPTFF by increasing the LMH in order to keep the volume flow constant, which is described in more detail in the batch process section. With this strategy, a realistic fluctuation of ±10% can be equalized. A controller based on UV detection for the breakthrough during loading maintains the switching times of the PCC so that the loading of the columns is optimized. Assuming a constant concentration, this means an additional 10% of product to be purified in the PCC, which is within the process design margin of the PCC. Any further increase would need to be buffered by a surge tank in front of the PCC to be processed when the flow rate decreases again. A decrease of 10% product mass would lead to a longer loading cycle in the PCC, which should also be within the specification.

The same logic applies to a deviation of product concentration of ±10% in the USP in this case, the fluctuation is controlled by the PCC by reducing the loading time. Any further increase in concentration will lead to a decrease in PCC loading time and therefore to scheduling issue, where the next column to be loaded has not yet finished the re-equilibration step. At this point, the feed needs to be diverted to a surge tank to be processed when the USP concentration is decreasing again. A reduction of VLP concentration will be controlled by increasing the loading time in the PCC, as described by Löfgren et al.^132,138^

For purity of the final product, the concentration of DNA in the perfusate is considered. As described in the batch control strategy, the developed process is robust enough to handle a deviation up to +600%. Unlike in the batch mode, additional processing time in the SPTFF is not possible in a continuous process; in the unlikely case of a DNA contamination, the product would need to be purified by an additional batch UFDF.

## 4 Discussion and Conclusions

Perfusion experiments operated with tubular depth filters in the ATF mode showed a very low filter capacity of less than 50 L/m^2^. Experiments with two layers of normal flow depth filters, covering the same cutoff range as the tubular filters, and analysis by filter blocking laws revealed that in ATF mode, the filter is blocked by particles, mainly cell debris, in the size range between 700 nm and 5 μm.

To confirm that the cause of low filter capacity is insufficient particle removal by alternating tangential flow, a harvest with the same filter module (2–5 μm cutoff) but by TFF instead of ATF using a low shear membrane pump was performed, which increased the filter capacity from 50 to 266 L/m^2^. As multihead membrane pumps like the Quattroflow series are already widely established in biomanufacturing for operations like UF/DF, this solution is easy to implement for industrial scale-up and can be used for fed-batch harvest as well as perfusion.

The proposed DSP is in contrast to existing processes fully integrated, closed, and continuous. Product isolation and factor 5 concentration increase is performed by continuous UF/DF using SPTFF technology and 300 kDa hollow fiber membranes operated at 40 LMH. Further purification is carried out by AEX at load of 1 × 10^11^ VLP/mL_ads_ and final UF/DF with performance parameters equal to UF/DF1. Total process yield is 59% (factor 1.5–3 higher than literature) with 99% decrease of DNA (1.5 ng/doses) and protein, thereby ensuring regulatory demanded purity levels of less than 10 ng_DNA_/doses gaining product specification.

DTs support accelerated process design and development^131,139,140^ up to basic and detailed engineering including process control system configuration and enable among others an operator training simulator in combination with the existing process control system, and they are a well-established and beneficial procedure in petro-, basic-, and fine-chemicals industry. Moreover, operator workload is reduced drastically, as they are enabled to operate different plants in parallel—a most wanted capacity increase option at enhanced product robustness.^79^ Digital-twin-based process automation reduces the number of operators required by a factor of 2 and lowers their workload and even stress level drastically.^131,141^

- DT + PAT-supported RTRT in DSP allows an increase in productivity in the DSP by a factor of 2 because hold times are eliminated.^84^
- Batch failures can be significantly reduced with the help of DTs; APC offers 99.9% reliability.
- In the USP, feeding can be optimized by mapping the metabolism, which, as shown here, can reproduce all significant experimental process parameters. In this way, the feeding time and quantity can be regulated based on the consumption of significant amino acids in order to increase productivity by up to 70%.
- By implementing optimized control strategies using PID controllers, CPP in the USP such as the pH value and the dissolved oxygen concentration can be precisely controlled. In addition, disturbance variables such as fluctuating volume flows, in particular the base, can be controlled, and thus the KPAs can be kept within the ideal range.
- By using such control strategies, batch failure rates can be lowered and process fluctuations, which can lead to a change in product concentration and quality, can be reduced.
- The continuous process operation in the USP, in combination with fed batch and/or perfusion, enables an increase in STY in cultivation by a factor of 9.6–10.9 compared to fed batch.
- A suitable cell retention system is required for this. Due to the size of the HI-VLPs, classic hollow fiber modules are not suitable. Alternatively, a combination of tangential flow and depth filtration can be used for cell retention in lentivirus production.
- ATF and TFF operation were examined. No particles >700 nm are present in the permeate. However, 10% of all particles in the bioreactor are in the size range of 0.7–5 μm, which is consistent with HEK-VLP cultivations from the literature. With the ATF, the product flow rate already collapses after approximately 17 L/m^2^, and a maximum filter capacity of 50 L/m^2^ like in the batchwise harvest via depth filtration is reached, whereas in TFF operation, the product permeability is given for >266 L/m^2^. The filter capacity , and the existing blocking mechanism of the ATF corresponds to those of the comparable depth filtration. This suggests that due to the geometry of the filter, in contrast to classic hollow fiber modules, there is no or hardly any backflow of the permeate, which prevents the membrane from blocking. Consequently, the cell broth is mainly filtered in direct flow.
- In order to exploit the potential of increasing STY through continuous cultivation, operation in TFF with a higher recirculation rate is preferable to ATF operation for stable gene expression for the production of HI-VLPs in HEK293 cells.

The benefits of DTs together with a QbD-based control strategy including PAT concept for mainly schedule optimization in VLP manufacturing have already been demonstrated in Hengelbrock et al., highlighting a productivity increase up to a factor of 2.^84^ Similarly, for other entities like pDNA,^142^ the advantages by applying a DT and APC strategy have proven additional productivity gains of 20% at 99.9% reliability. The elimination of OOS batches as well as the opportunity of RTRT as additional benefits have been discussed in ref (79) as it has the effort of about 4 scientists developing over 2–3 weeks, if experienced, trained, and skilled in laboratory work, process modeling, and PAT with PCS. Final validation with manufacturing data runs would take about 1–2 months studies based on the DT, as an educated guess. The authors offer accessibility to companies of interest or further studies to overcome potential obstacles for industrialization of DT technology.
